# Effects of SZV-2649, a new multiple ion channel inhibitor mexiletine analogue

**DOI:** 10.1038/s41598-024-73576-5

**Published:** 2024-10-05

**Authors:** Aiman Saleh A. Mohammed, Muhammad Naveed, Tamara Szabados, István Szatmári, Bálint Lőrinczi, Péter Mátyus, Andrea Czompa, Péter Orvos, Zoltán Husti, Tibor Hornyik, Leila Topal, Szilvia Déri, Norbert Jost, László Virág, Péter Bencsik, István Baczkó, András Varró

**Affiliations:** 1 Department of Pharmacology and Pharmacotherapy, Albert Szent-Györgyi Medical School, University of Szeged, Szeged, Hungary; 2 Institute of Pharmaceutical Chemistry, Faculty of Pharmacy, University of Szeged, Szeged, Hungary; 3 HUN-REN-SZTE Stereochemistry Research Group, Hungarian Research Network, Szeged, Hungary; 4 Department of Organic Chemistry, Semmelweis University, Budapest, Hungary; 5 National Laboratory of Infectious Animal Diseases, Antimicrobial Resistance, Veterinary Public Health and Food Chain Safety, University of Veterinary Medicine, Budapest, Hungary; 6 HUN-REN-SZTE Research Group for Cardiovascular Pharmacology, Hungarian Research Network, Szeged, Hungary; 7 Interdisciplinary Research and Development and Innovation Centre of Excellence, University of Szeged, Szeged, Hungary

**Keywords:** Cardiology, Cardiovascular biology, Cardiovascular diseases, Arrhythmias, Atrial fibrillation, Ventricular fibrillation, Ventricular tachycardia

## Abstract

The antiarrhythmic and cardiac electrophysiological effects of SZV-2649 that contains a 2,6-diiodophenoxy moiety but lacks the benzofuran ring system present in amiodarone, were studied in mammalian cell line, rat and dog cardiac preparations. SZV-2649 exerted antiarrhythmic effects against coronary artery occlusion/reperfusion induced ventricular arrhythmias in rats and in acetylcholine- and burst stimulation induced atrial fibrillation in dogs. SZV-2649 inhibited hERG and GIRK currents in HEK cells (IC_50_: 342 and 529 nM, respectively). In canine ventricular myocytes, SZV-2649 (10 µM) decreased the densities of I_Kr_, and I_to_ outward and I_NaL_ and I_CaL_ inward currents. The compound (2.5–10 µM) elicited Class IB type V_max_ reducing and Class III type action potential duration prolonging effects in dog right ventricular muscle preparations. In canine atrial muscle, SZV-2629 (2.5–10 µM) moderately prolonged action potential duration and this effect was greatly augmented in preparations pretreated with 1 µM carbachol. In conclusion, SZV-2649, has antiarrhythmic effects based on its multiple ion channel blocking properties. Since its chemical structure substantially differs from that of amiodarone, it is expected that SZV-2649 would exhibit fewer adverse effects than the currently used most effective multichannel inhibitor drug amiodarone and may be a promising molecule for further development.

**Supplementary Information:**

The online version contains supplementary material available at 10.1038/s41598-024-73576-5.

## Introduction

Cardiovascular diseases including sudden cardiac death and stroke are among the leading causes of mortality in industrialized countries. The most serious ventricular arrhythmia - ventricular fibrillation (VF) - causes more than 300 000 deaths in the USA annually^1^. Atrial fibrillation (AF) is the most common chronic arrhythmia with 2-5 % incidence in the elderly (60–70 year old) population^2^. In addition, AF can lead to severe or life-threatening ventricular arrhythmias including VF^3^ and it is also one of the most important contributors to the pathogenesis of stroke^2^. At present, the pharmacological management of arrhythmias including VF and AF is not satisfactory, since the available drugs either do not control arrhythmias properly or induce serious side effects^4^. Therefore, there is an increasing demand for safer and more effective new compounds to treat and prevent AF and ventricular arrhythmias as well.

The available Class III antiarrhythmic drugs like dofetilide, ibutilide or sotalol and Class IC drugs like flecainide or propafenone, which are currently used drugs in the field, have substantial proarrhythmic potential as shown in the CAST, CASH and SWORD studies^5–7^, greatly limiting their clinical use. Unfortunately, the proarrhythmic and antiarrhythmic mechanisms are closely related to each other i.e., in the case of Class III antiarrhythmic effect, the reverse rate-dependent repolarization prolonging effect, and in the case of Class IC antiarrhythmic effect, the sodium channel blocking effect with slow kinetical recovery.

To avoid this problem, new compounds like XEN-D0103 or NS8593 were developed^8,9^ in recent years to treat AF by selective inhibition of certain potassium channels (Kv1.5 and SK2) that are not or weakly expressed in ventricular muscle^10,11^ but are abundant in the atria^10,12^. These drugs seem to have no or fewer proarrhythmic side effects, however, serious questions emerged regarding their therapeutical efficacy^4,13^.

The application of amiodarone still represents one of the most effective pharmacological treatment options to manage AF and ventricular arrhythmias^14,15^. Amiodarone has lower proarrhythmic risk than other currently used antiarrhythmics^16,17^. However, it exerts serious extracardiac adverse effects like pulmonary fibrosis, hepatotoxicity, photodermatosis, cornea deposition, thyroid dysfunction, which greatly limit its clinical use^18^. In addition, the toxic effect of amiodarone is further complicated by its slow elimination (half-life of 60–80 days or more during long-term therapy! ) resulting in drug accumulation in different tissues of the body. During chronic amiodarone treatment an electrophysiologically active metabolite, desethylamiodarone – that has similar chemical structure and electrophysiological effects - appears in the plasma and also accumulates in various tissues including the heart^19–22^. Amiodarone has a very complex mode of action inhibiting cardiac sodium, calcium, potassium currents and β-adrenergic receptors^15,23,24^. It has a benzofurane structure and also contains iodine. Therefore, it is generally thought that both amiodarone and desethylamiodarone inhibit and interfere^25–28^ with cardiac thyroid receptors and it is possible that they exert their antiarrhythmic effect partly by this mechanism. On the other hand, the benzofuran structure can be responsible for some of the serious adverse effects also seen with the less effective, but iodine free analogue, dronedarone^4,29–31^.

**Fig. 1:**
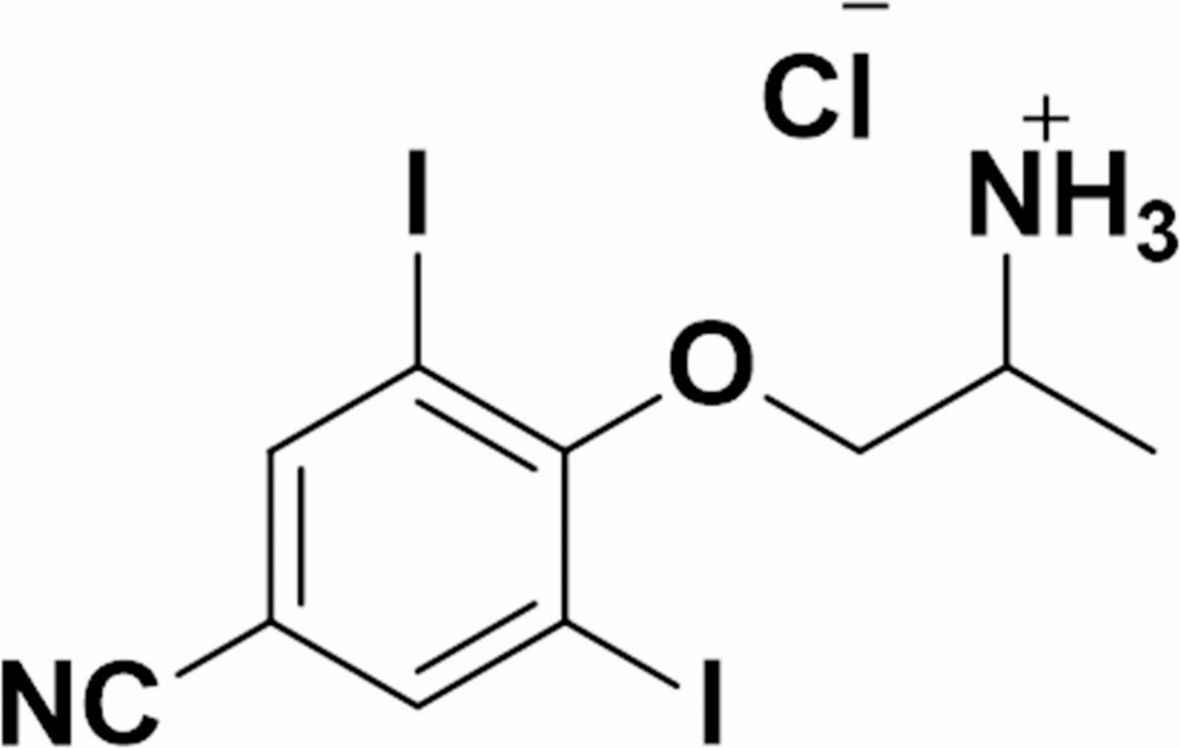
Chemical structure of SZV-2649 [4-(2-aminopropoxy)-3,5-diiodobenzonitrile hydrochloride].

Therefore, the aim of our project was to develop drug candidates which possess complex electrophysiological effects similar to those of amiodarone while exhibiting fewer adverse effects than amiodarone. As a continuation of our pioneering approach to design conceptually novel dual-mode antiarrhythmic agents based on our 3D QSAR modeling studies^32–35^, we synthesized SZV-2649 (Fig. 1) and a limited number of its analogs, which formed the basis of a recently filed patent application (P2300429/10)^36^. The effects of the most promising compound of this series, SZV-2649, are described in the present paper. SZV-2649 shows multiple cardiac ion channel inhibitory properties similar to those of amiodarone/desethyamiodarone, while having a distinctly different chemical structure, in particular, lacking the benzofuran moiety of amiodarone. By this approach, it can be expected that this novel compound, SZV-2649, compared to amiodarone, would have distinctly different pharmacokinetics and a less disadvantageous adverse effect profile.

## Results

### Effects of SZV-2649 on hERG and GIRK channels

Inhibition of the hERG channel, the pore forming α-subunit of the native I_Kr_ cardiac potassium channel, is expected to prolong repolarization both in the atria and in the ventricles. The GIRK channel is the pore forming α-subunit of the acetylcholine-activated potassium channel and plays an important role in atrial electrophysiology. Its inhibition can be expected to decrease the acetylcholine-activated potassium current (I_KACh_), a current that plays an important role in the pathogenesis of AF. Therefore, the effects of SZV-2649 on the hERG and GIRK ion channels were studied by the planar patch-clamp technique in mammalian HEK cell lines stably expressing these channels.

**Fig. 2:**
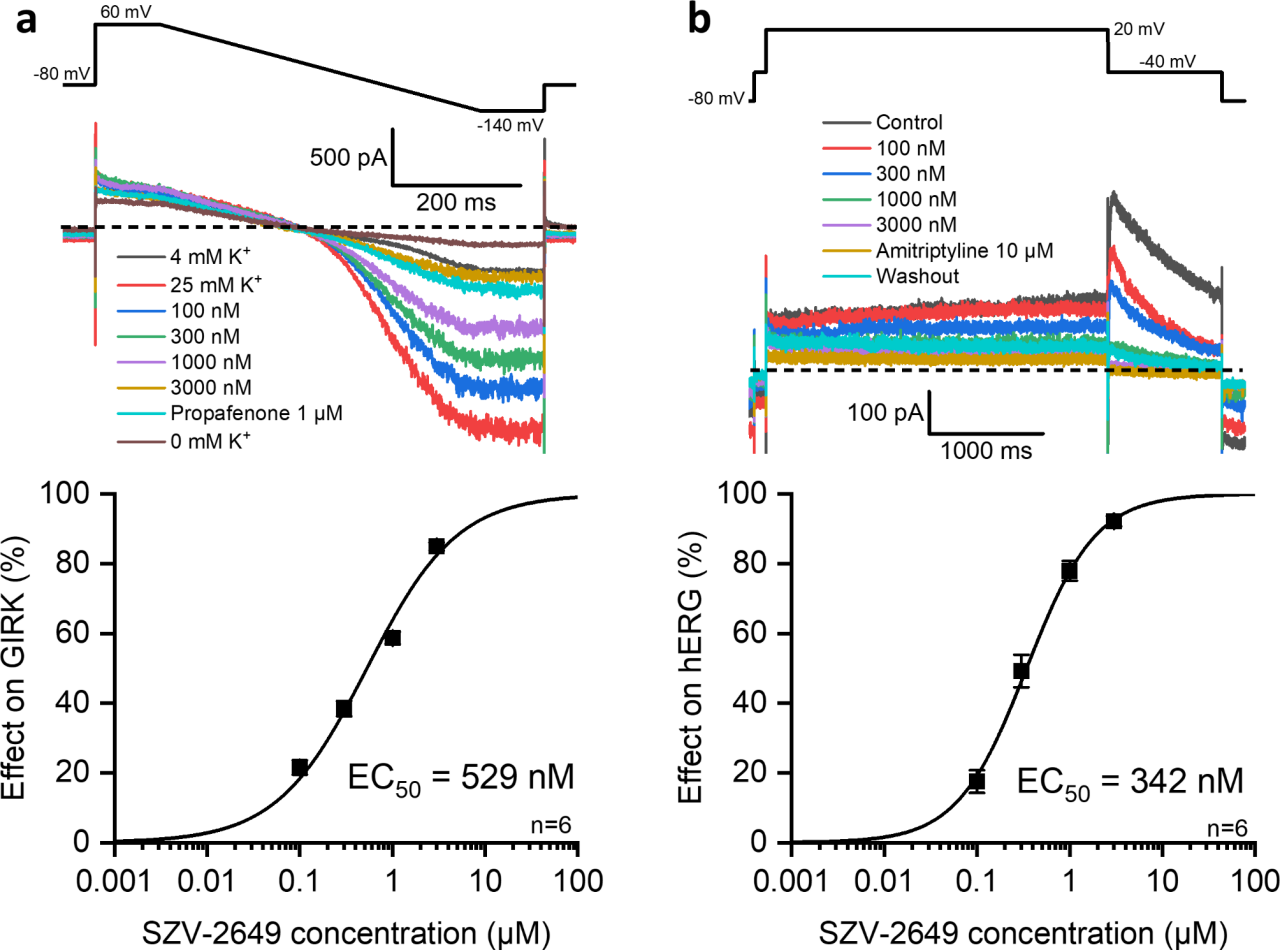
Effects of SZV-2649 on GIRK (**a**) and on hERG (**b**) currents in HEK cell lines. *Top* panels display original recordings of GIRG (**a**) and hERG (**b**) currents treated with 100 nM, 300 nM, 1000 nM and 3000 nM SZV-2649. Propafenone (1 µM) and amitriptyline (10 µM) were applied as reference inhibitors for GIRK (**a**) and hERG (**b**), respectively. Insets show the voltage protocols. *Bottom* panels display concentration-response curves of SZV-2649 inhibitory activity on GIRK (**a**) and hERG (**b**) channels. The dashed lines indicate zero current levels. Data are expressed as means ± SEM.

As Fig. 2A shows, SZV-2649 strongly inhibited the GIRK channel in the low micromolar concentration range with an IC_50_ of 0.529 µM. The magnitude of its effect on the hERG channel was similar and could be characterized with an IC_50_ value of 0.342 µM (Fig. 2B).

## The effect of SZV-2649 on transmembrane ionic currents in dog ventricular myocytes

The effects of SZV-2649 on transmemrane ionic currents were investigated in dog cardiac myocytes after a 5-minute superfusion of the drug.

**Fig. 3:**
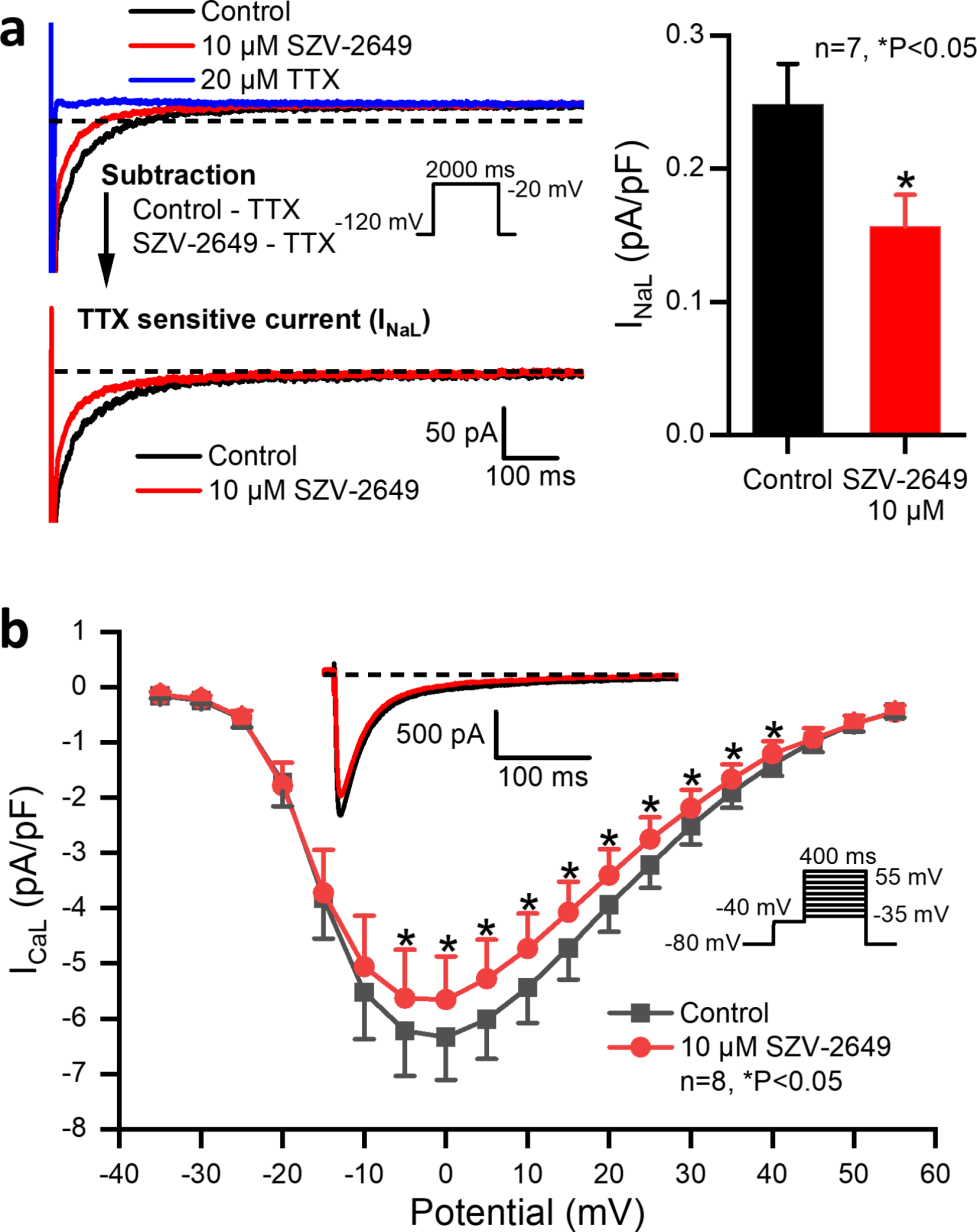
Effect of SZV-2649 on the late sodium (I_NaL_) and L-type calcium (I_CaL_) currents in dog left ventricular myocytes. Panel **a** indicates the effects of 10 µM SZV-2649 administration on the I_NaL_ current showing original current recordings (*left*) and bar diagrams (*right*). Panel **b** displays current-voltage relationship of I_CaL_ recorded with 3000 ms pulsing cycle length in control conditions and in the presence of 10 µM SZV-2649. Inset (*top*) shows original current records in control conditions and after application of 10 µM SZV-2649. The dashed lines indicate zero current levels. The applied voltage protocols are displayed on the insets. Data are expressed as means ± SEM. *P < 0.05 vs. control.

The late sodium current (I_NaL_) was investigated by application of 2-second-long test pulses to -20 mV from the holding potential of -120 mV with the pulsing cycle length of 5 s. In the internal (pipette) solution, KCl was replaced by 125 mM CsCl to block potassium currents and the external solution contained 1 µM nisoldipine, 0.5 µM HMR-1556 and 0.1 µM dofetilide in order to block I_CaL_, I_Ks_ and I_Kr_ currents, respectively. As Fig. 3A indicates, SZV-2649 significantly decreased the magnitude of I_NaL_.

The L-type inward calcium current (I_CaL_) was measured from a holding potential of -80 mV with a 40 ms prepulse to -40 mV to inactivate I_Na_ (see inset of Fig. 3B). Test pulses of 400 ms duration from − 35 mV to 55 mV were applied with a pulsing cycle length of 3 s. In the internal (pipette) solution, KCl was replaced by 125 mM CsCl to block potassium currents. As Fig. 3B shows, SZV-2649 moderately but significantly decreased the magnitude of I_CaL_.

**Fig. 4:**
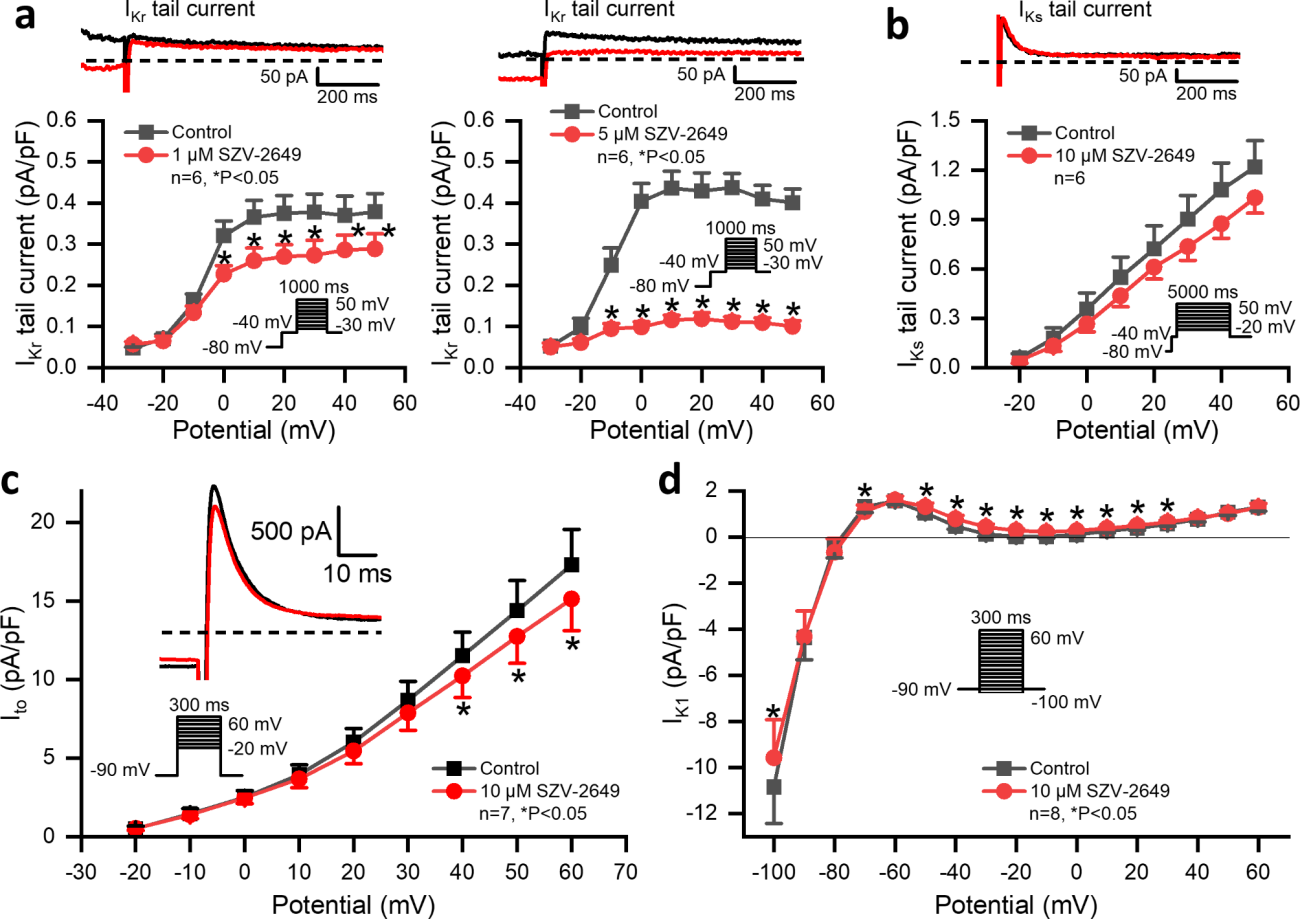
Effect of SZV-2649 on the rapid (I_Kr_) and slow (I_Ks_) delayed rectifier, the transient outward (I_to_) potassium currents and on the steady state current-voltage relationship in dog left ventricular myocytes. *Top* panels display the tail current section of original I_Kr_ (**a**) and I_Ks_ (**b**) current traces in control conditions and in the presence of SZV-2649. Dashed lines refer to the baseline for I_Kr_ and I_Ks_ tail current levels after the test pulse at -40 mV. *Bottom* panels illustrates current-volatage relationship for I_Kr_ (**a**) and I_Ks_ (**b**). SZV-2649 was applied at concentrations of 1 µM and 5 µM for I_Kr_ and 10 µM for I_Ks_ current. Panel **c** indicates current-voltage relationship of I_to_ recorded with 3000 ms pulsing cycle length in control conditions and in the presence of 10 µM SZV-2649. Original current records in control conditions and after application of 10 µM SZV-2649 are displayed on the inset. Dashed lines refer to the zero current level. Panel **d** shows the steady state current-voltage relation recorded with 3000 ms pulsing cycle length in control conditions and in the presence of 10 µM SZV-2649. The applied voltage protocols are displayed on the inset. Data are expressed as means ± SEM. *P < 0.05 vs. control.

The rapid delayed rectifier potassium current (I_Kr_) was elicited from the holding potential of -80 mV following a prepulse to -40 mV. I_Kr_ tail current was measured at a post pulse to -40 mV after 1 s long test pulses from − 30 mV to 50 mV using 10 mV increments at the pulsing cycle length of 20 s (see inset of Fig. 4A). The effect of SZV-2649 was investigated at 1 and 5 µM concentrations. As Fig. 4A indicates, SZV-2649 inhibited I_Kr_ markedly and significantly by approximately 23% and 75% at 1 and 5 µM concentrations, respectively.

The slow delayed rectifier potassium current (I_Ks_) was elicited from the holding potential of -80 mV with a prepulse to -40 mV. The I_Ks_ tail current was measured at a post pulse to -40 mV after 5-second-long test pulses from − 20 mV to 50 mV using 10 mV increments at the pulsing cycle length of 10 s (see inset of Fig. 4B). The effect of SZV-2649 was investigated at the concentration of 10 µM. As Fig. 4B shows, SZV-2649 did not change or only minimally decreased the amplitude of the I_Ks_ tail current.

The effect of SZV-2649 on the transient outward potassium current (I_to_) was investigated at 10 µM concentration with 300-millisecond-long test pulses from − 20 mV to 60 mV using 10 mV increments from the holdig potential of -90 mV at the pulsing cycle lenth of 3 s (see inset of Fig. 4C). The difference between the peak current at the beginning and at the end of the test pulses was defined as I_to_. As Fig. 4C shows, SZV-2649 did not or only minimally decreased the amplitude of I_to_.

Figure 4D represents the steady state current-voltage relation after 300 ms pulses from − 100 mV to 60 mV using 10 mV increments applying 3-second pulsing cycle length from the holding potential of -90 mV. SZV-2649 did not significantly change the current-voltage relation except in the voltage range between − 50 mV to 30 mV. This suggests that SZV-2649 has no discernible effects on the inward rectifier outward potassium (I_K1_) current, and any possibly depolarization activated steady state currents. The positive shift of the current-voltage curve between − 50 mV and 30 mV can be attributed to the inhibition of the I_NaL_, or non inactivating “window”, or background sodium current.

## The effects of SZV-2649 on dog cardiac action potentials

The effects of SZV-2649 on action potentials were studied in dog ventricular, atrial and Purkinje fiber preparations. Results at the physiologic stimulation frequency (60 beats/minute = 1 Hz) are shown in Table 1. As shown in Table 1, SZV-2649 decreased V_max_ and prolonged APD_90_ in ventricular muscle and atrial preparations in a concentration-dependent manner, indicating Class I and Class III antiarrhythmic properties of the compound. In Purkinje fiber preparations, SZV-2649 significantly shortened APD_50_ but did not significantly prolong APD_90_, suggesting no increased dispersion of repolarization and refractoriness between ventricular muscle and Purkinje fibers.

**Table 1:** The effect of SZV-2649 on the major action potential parameters in ventricular and atrial muscle and in Purkinje fibers. CL, stimulation cycle length; RP, resting membrane potential; APA, action potential amplitude; V_max_, maximum upstroke velocity, APD_50_ and APD_90_ action potential duration measured at 50% and 90% of repolarization.  *P < 0.05 vs. control.

		RP (mV)	APA (mV)	V_max_ (V/s)	APD_50_ (ms)	APD_90_ (ms)
**Ventricular muscle (n = 8)** **CL = 1000 ms**	**Control**	-86.4 ± 1.1	118.0 ± 2.6	240.2 ± 21.3	190.2 ± 3.2	227.1 ± 3.3
	**SZV-2649** **2.5 µM**	-84.6 ± 1.3	116.8 ± 2.7	228.3 ± 23.5	204.2 ± 4.5*	240.0 ± 3.5*
	**SZV-2649** **5 µM**	−84.5 ± 2.0	118.3 ± 1.9	227.4 ± 24.2	212.2 ± 5.7*	251.6 ± 4.0*
	**SZV-2649** **10 µM**	−86.1 ± 2.0	117.1 ± 2.1	203.2 ± 24.3*	213.4 ± 5.8*	252.8 ± 5.0*
**Atrial muscle (n= 11)** **CL = 1000 ms**	**Control**	−87.7 ± 1.5	109.4 ± 2.1	205.8 ± 17.0	75.6 ± 7.3	160.8 ± 9.1
	**SZV-2649** **5 µM**	−88.2 ± 1.8	108.8 ± 1.9	195.3 ± 22.0	73.3 ± 6.2	168.9 ± 8.8*
	**SZV-2649** **10 µM**	−85.1 ± 1.7	102.6 ± 3.2*	145.9 ± 12.1*	70.1 ± 6.2	176.8 ± 8.5*
**Purkinje fiber (n = 10)** **CL = 500 ms**	**Control**	−89.2 ± 2.1	127.4 ± 2.2	633.4 ± 37.3	144.8 ± 4.2	216.3 ± 5.0
	**SZV-2649** **2.5 µM**	−89.3 ± 2.0	128.8 ± 1.5	581.1 ± 39.0	128.9 ± 6.0*	219.4 ± 3.7
	**SZV-2649** **5 µM**	−84.7 ± 1.7	123.8 ± 2.3	507.4 ± 53.4*	113.6 ± 5.6*	217.4 ± 5.1
	**SZV-2649** **10 µM**	−86.0 ± 1.1	119.2 ± 3.3	415.0 ± 28.9*	112.4 ± 7.9*	223.4 ± 5.6

**Fig. 5:**
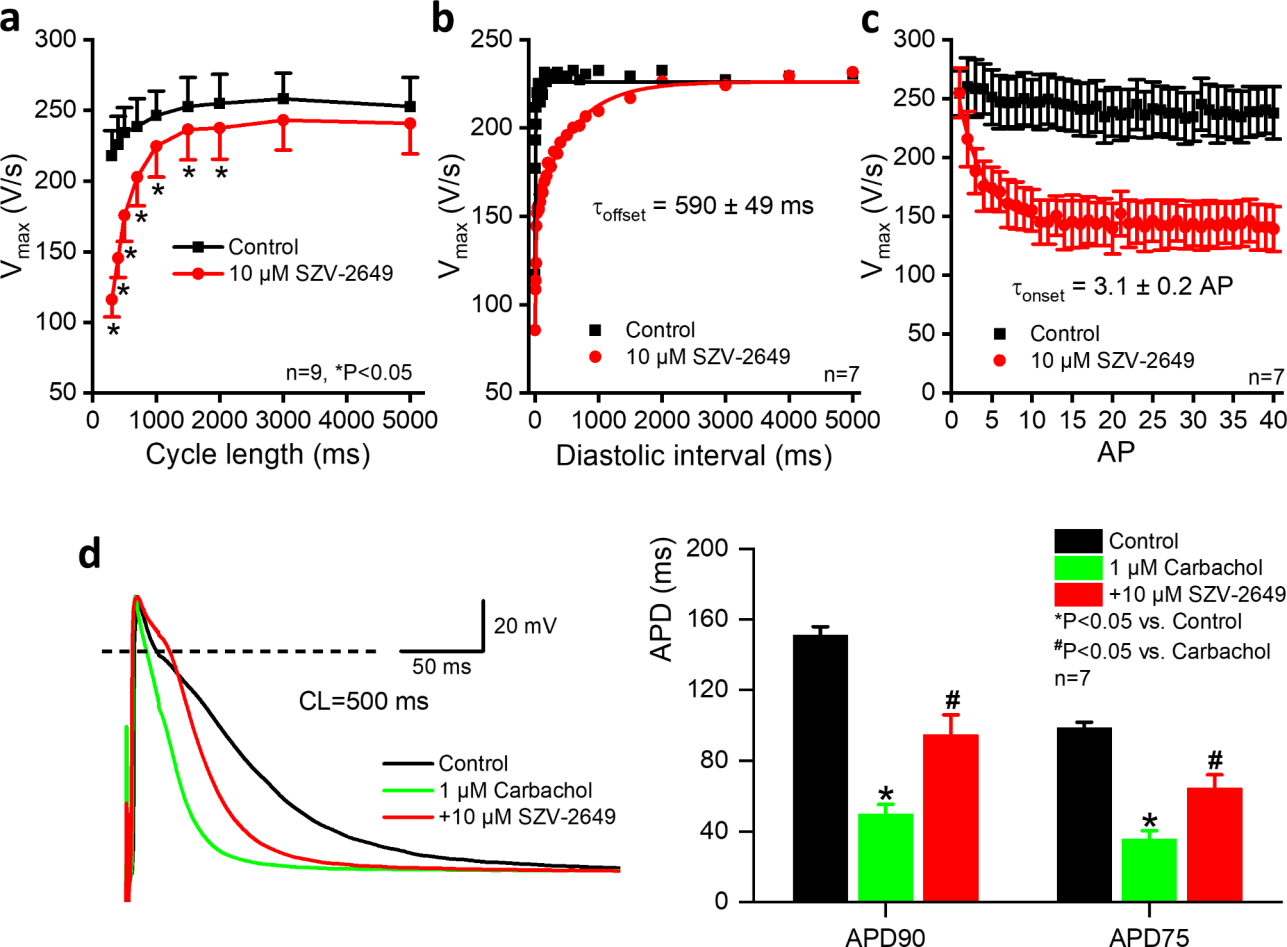
Effect of SZV-2649 on the maximum upstroke velocity (V_max_) of action potential in dog ventricular muscle and on the action potential duration (APD) in atrial preparations. Panel **a** indicates the effect of 10 µM SZV-2649 on V_max_ as a function of stimulation frequency. Panel **b** shows the recovery (“offset”) kinetics of SZV-2649-elicited V_max_ block, while panel **c** displays the onset kinetics of use-dependent V_max_ block after 10 µM SZV-2649 administration. Panel **d** indicates the effect of SZV-2649 on APD in the presence of carbachol in dog atrial trabecular muscle. Effects of 10 µM SZV-2649 administration on action potential after application of 1 µM carbachol are indicated showing original records (*left*) and bar diagrams (*right*). The dashed line indicates zero potential level. APD_90_, action potential duration at 90% of repolarization; APD_75_, action potential duration at 75% of repolarization. Data are expressed as means ± SEM. *P < 0.05 vs. control; #*P* < 0.05 vs. carbachol.

The Class I antiarrhythmic property of SZV-2649 was further studied with different frequency-dependent protocols. In this study, different steady-state stimulation cycle lengths were applied - like those previously published with amiodarone^37^. SZV-2649 decreased V_max_ at cycle lengths shorter than 2000 ms (Fig. 5A). Also, the recovery of V_max_ was measured (offset kinetics of V_max_ block) with premature stimulation with gradually increasing diastolic intervals from 1 Hz basic frequency, and the recovery time constant was 590 ms (Fig. 5B), very similar to that observed previously following chronic amiodarone application^38^. The onset kinetics of V_max_ block, i.e. the development of V_max_ inhibition after 1 min rest at the stimulation cycle length of 400 ms was 3.1, that is relatively fast (Fig. 5C), similar to that of previously found^38^ with amiodarone. Based on the observed frequency-dependent V_max_ inhibitory behavior, the effect of SZV-2649 – like that of amiodarone – can be characterized as showing Class I/B antiarrhythmic properties.

The possible effect of SZV-2649 on I_KACh_ was investigated in dog atrial trabecular muscle preparations. Carbachol (1 µM) was added to the tissue bath for 10 min to activate I_KACh_. As Fig. 5D shows carbachol substantially shortened atrial repolarization. Application of 10 µM SZV-2649 prolonged APD substantially to a greater degree (100%) than could be expected by its effect on repolarization in the absence of carbachol (10%) as shown in Table 1; Fig. 5D.

## Effect of SZV-2649 on the basic ECG parameters in conscious dogs

The effects of SZV-2649 on the basic ECG parameters in conscious dogs were investigated. The orally and intravenously applied SZV-2649 (10 mg/kg), as Table 2 indicates, decreased heart rate (increased the RR interval), increased the PQ interval and prolonged repolarization indicated by the increase in the frequency corrected QT interval (QT_c_).

**Table 2:** The effect of orally (1 h after administration) and intravenously (30 min after administration) applied SZV-2649 (10 mg/kg) on the basic ECG parameters in conscious dogs. *P< 0.05 vs. control.

	RR (ms)	PQ (ms)	QRS (ms)	QT (ms)	QT_C_ (ms)
*per os 10 mg/kg* (*n* = 7)					
**Control**	586.7 ± 39.3	113.9 ± 7.3	48.3 ± 4.3	197.8 ± 4.1	233.7 ± 2.7
**SZV-2649**	596.2 ± 44.0	119.8 ± 7.5*	47.9 ± 2.4	215.0 ± 7.5	250.1 ± 7.0*
***Intravenous 10 mg/kg (n = 6)***					
**Control**	588.9 ± 38.1	119.1 ± 8.7	50.2 ± 3.5	208.1 ± 5.0	243.9 ± 6.0
**SZV-2649**	756.1 ± 29.4*	150.6 ± 15.8	52.2 ± 3.2	234.5 ± 5.6*	255.7 ± 6.3*

### Effects of SZV-2649 on ventricular arrhythmias in anesthetized rats after coronary occlusion/reperfusion

**Fig. 6:**
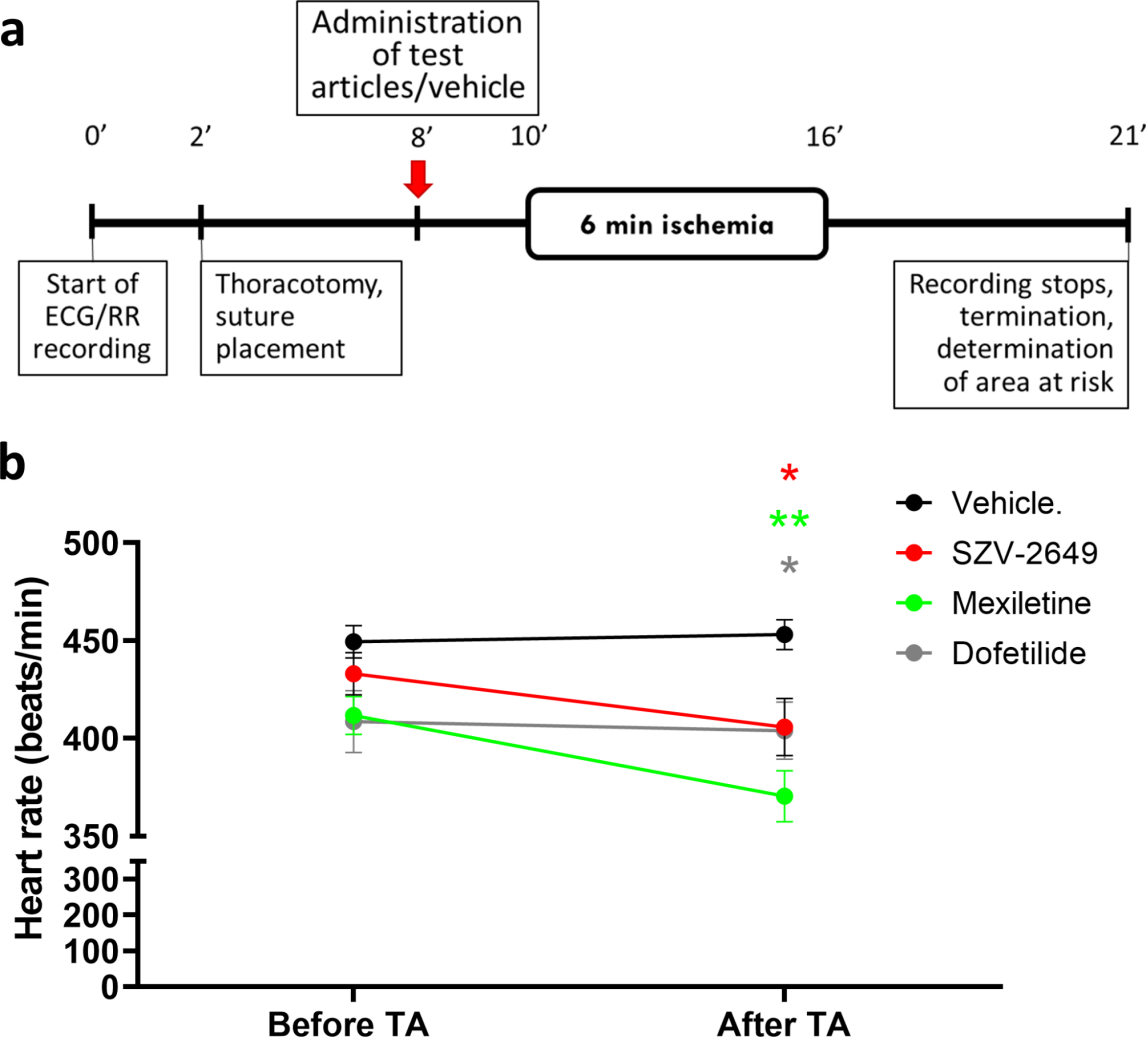
Experimental protocol for the in vivo test system to assess ischaemia-evoked arrhythmias in anesthetized rats following treatment with the test articles (**a**). Heart rates before and after the administration of test articles (TA) in anesthetized rats subjected to coronary artery occlusion/reperfusion (**b**). Data are expressed as mean ± S.E.M., *n* = 8–11, *P < 0.05, **P < 0.001 as analyzed by repeated measures two-way ANOVA with Tukey’s multiple comparisons post hoc test as compared to Vehicle.

**Fig. 7:**
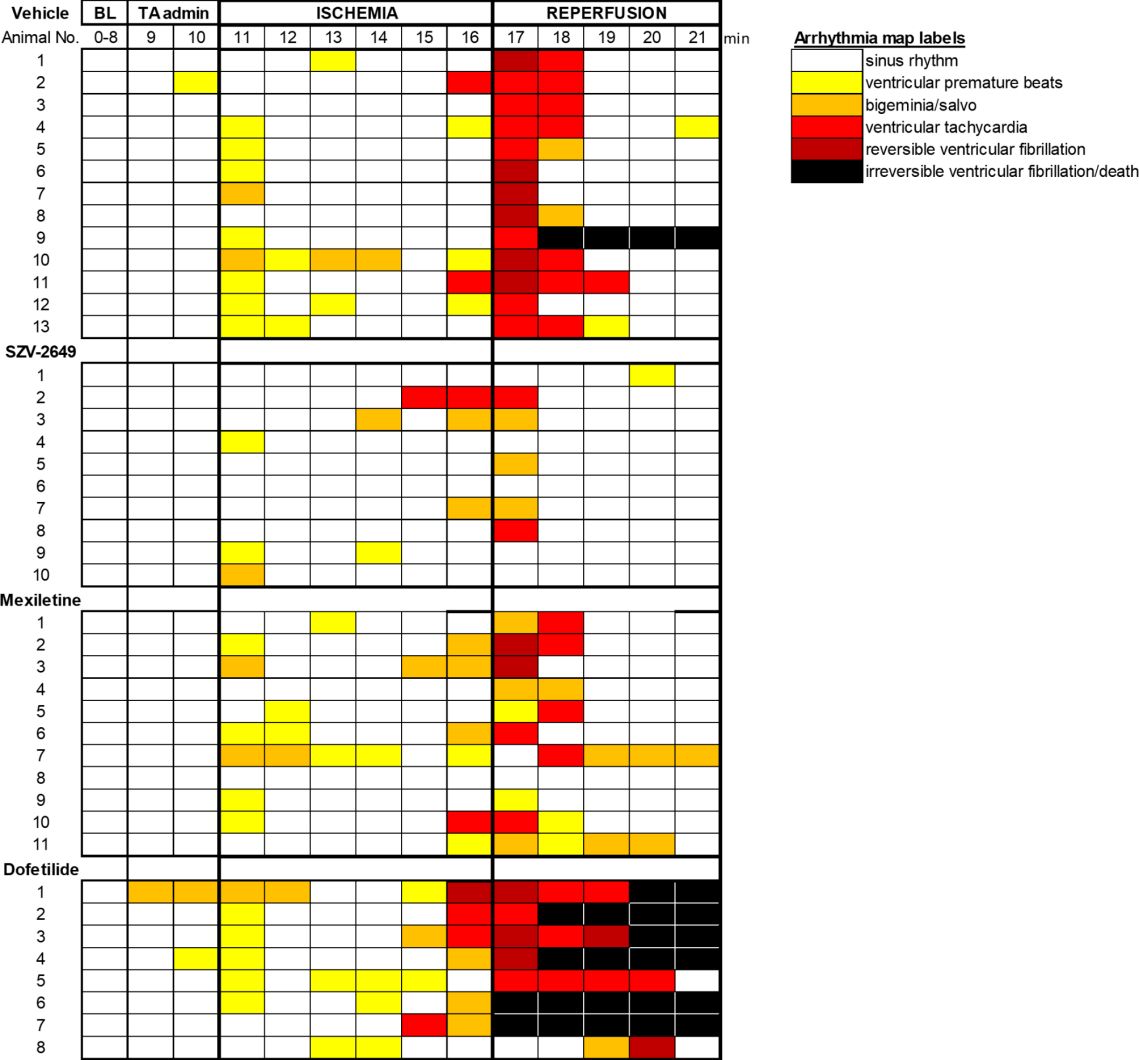
Detailed arrhythmia map showing the arrhythmias in the order of severity during 6 min ischemia followed by 5 min of reperfusion in anesthetized rats induced by coronary artery occlusion and its release, respectively. Each row represents arrhythmias of each animal. The different color boxes show 1 min periods. BL, baseline; TA, test article.

**Fig. 8:**
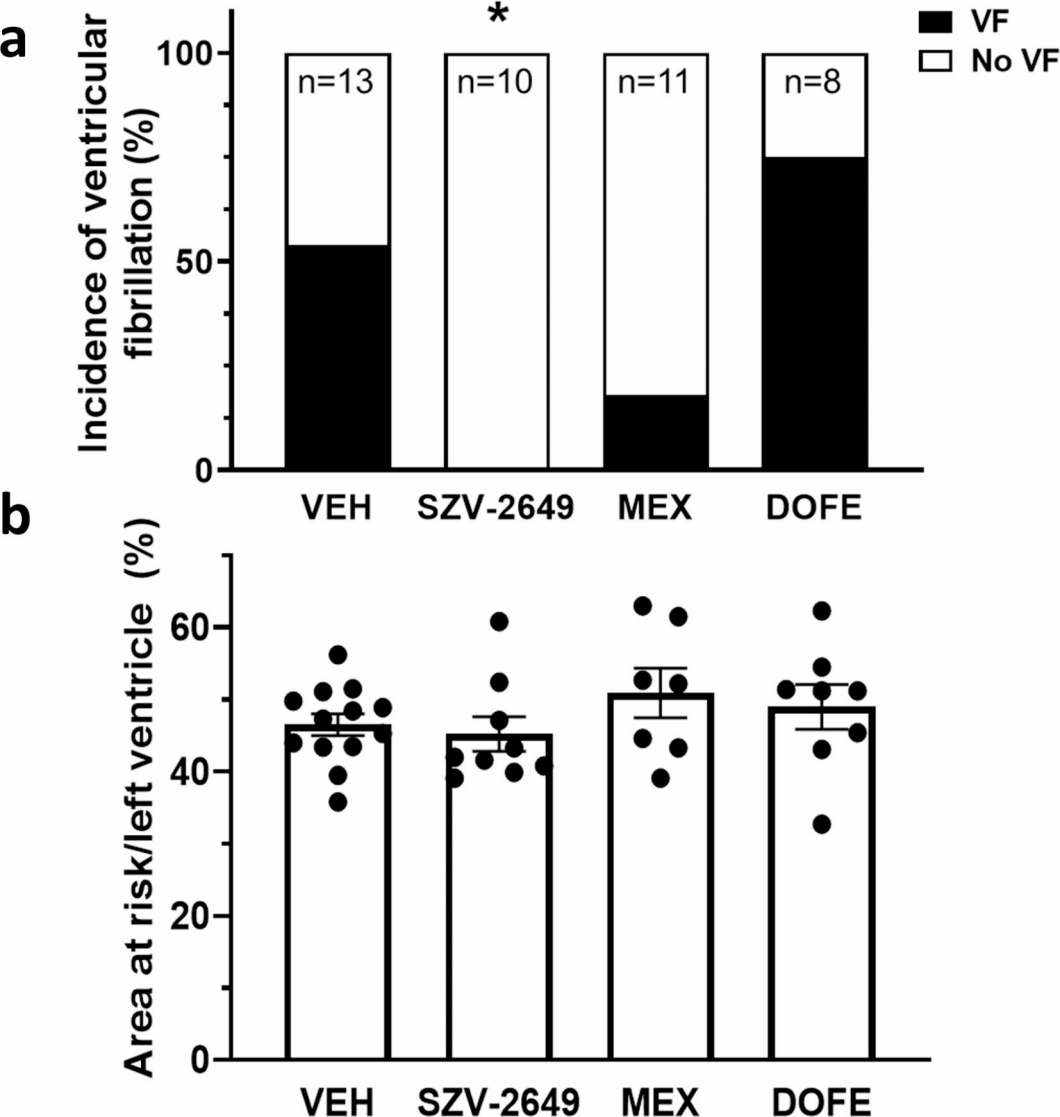
Grouped data showing the incidence of coronary artery occlusion/reperfusion induced ventricular fibrillation (VF) **(a)** and myocardial area at risk **(b)** in rats. *n* = 9–14, *P < 0.05 as analyzed by Chi-square test with Yate’s correction, pairwise compared to Vehicle (VEH, Vehicle; MEX, mexiletine; DOFE, dofetilide).

The effects of SZV-2649 were further studied in the acute ischemia-induced ventricular arrhythmia model in anasthetized rats. Mexiletine and dofetilide were used as reference compounds for comparison. In anesthetized rats, SZV-2649 and both reference compounds, mexiletine and dofetilide, significantly decreased heart rate following *i.v.* administration (Fig. 6B). Importantly, as shown on Fig. 7, SZV-2649 markedly reduced the incidence of ventricular arrhythmias induced by coronary artery occlusion/reperfusion, while the reference compound mexiletine also showed some antiarrhythmic effects, however, the I_Kr_ blocker dofetilide increased the incidence of arrhythmias in this model. As grouped data on Fig. 8 shows, ventricular fibrillation developed in over 50% of the control animals during reperfusion, while VF did not develop (0%) after application of SZV-2649. The effects of the reference drugs were less pronounced and non-significant, since mexiletine decreased the incidence of VF to 18%, while the application of dofetilide even further increased it to 75% (Fig. 8a). There were no significant differences in the ischemic areas of risk in the different groups (Fig. 8b).

### Effects of SZV-2649 on atrial fibrillation induced by carbachol infusion and electrical stimulation in anesthetized dogs

The effect of SZV-2649 was further investigated in carbachol-infused and programmed electrical stimulation-induced (i.e. 800/min atrial burst stimulation) acute atrial fibillation model in anesthetized dogs. In these experiments, propafenone was used as the reference compound. As indicated in Table 3, under the influence of carbachol, sustained atrial fibrillation was successfully induced with a burst stimulus both in the propafenone (i.v. 0.3 mg/kg) group and in the SZV-2649 (i.v. 10 mg/kg) group. Without the use of carbachol, it was not possible to induce sustained atrial fibrillation with burst stimulus alone. Similar to the propafenone group, as shown in Table 3, an intravenous bolus of 10 mg/kg SZV-2649 converted atrial fibrillation to sinus rhythm in 100% of experimental animals. Under the influence of propafenone and SZV-2649, atrial fibrillation was reinduced only 2 (1.6%) and 3 (2%) times, respectively, by the 25 burst stimuli delivered in each animal, further supporting the substantial protective effect of SZV-2649 against atrial fibrillation.

**Table 3:** Effects of SZV-2649 on atrial fibrillation induced by carbachol infusion and electrical stimulation in anesthetized dogs. Individual responses to interventions in each animal are indicated. N, response not observed; Y, response observed.

Experiment	Control burst without Carbachol	Carbachol 50 ug iv bolus + 100 µg/h maintenance + burst	After Propafenone or SZV-2649	After Propafenone or SZV-2649
Propafenon group (i.v. 0.3 mg/kg)	AF induced?	AF induced?	AF converted to SR?	AF reinduced?
DOG 1	N (-)	Y (+)	Y (+) 291 s	N (-)
DOG 2	N (-)	Y (+)	Y (+) 89 s	N (-)
DOG 3	N (-)	Y (+)	Y (+) 589 s	Y (+) 1/25
DOG 4	N (-)	Y (+)	Y (+) 15 s	Y (+) 1/25
DOG 5	N (-)	Y (+)	Y (+) 145 s	N (-)
*SZV-2649 group (i.v. 10 mg/kg)*				
DOG 1	Y(+)	Y (+)	Y (+) 79 s	N (-) 0/25
DOG 2	N (-)	Y (+)	Y (+) 175 s	N (-) 0/25
DOG 3	Y(+)	Y (+)	Y (+) 64 s	N (-) 0/25
DOG 4	N (-)	Y (+)	Y (+) 286 s	Y (+) 1/25
DOG 5	N (-)	Y (+)	Y (+) 84 s	Y (+) 2/25
DOG 6	N (-)	Y (+)	Y (+) 194 s	N (-) 0/25

## Discussion

The recently developed SZV-2649 is a member of a new limited number of patented iodophenoxypropane-2-amine compounds (P2300429/10). Its structure combines Class IB (mexiletine) and iodide (amiodarone) like structural elements. The purpose of the present experiments was to study the antiarrhythmic and cellular electrophysiological effects of SZV-2649 in experimental arrhythmia models, as well as in isolated cardiac tissue and cellular preparations, making the assumption that combining these structural elements would result in a promising new antiarrhythmic drug candidate. Accordingly, the main finding of the present study was that SZV-2649 exerted antiarrhythmic effects against both experimental ventricular and atrial arrhythmias. Based on the cellular electrophysiological experiments, the proposed mechanisms of the antiarrhythmic properties of SZV-2649 include the inhibition of the acetylcholine-activated and hERG/I_Kr_ outward potassium, and inward sodium and calcium currents. These effects on the investigated transmembrane ionic currents resulted in the prolongation of repolarization as well as the mexiletine/amiodarone-like (Class IB) inhibition of depolarization.

The therapeutic values of both selective Class I and Class III antiarrhythmic effects alone are not satisfactory, since several clinical studies showed dissapointing results with drugs like lidocaine, mexiletine, sotalol and dofetilide exhibiting either Class IB or Class III antiarrhythmic properties^4,39,40^. This disappointment could be attributed to the lack of proper effectiveness or due to their proarrhythmic adverse effects^41–43^. However, it is generally accepted that amiodarone has strong antiarrhythmic efficacy both against ventricular and atrial arrhythmias with relatively fewer proarrhythmic complications^15,44^. These properties were considered to relate to findings showing that amiodarone had multiple modes of action including Class IB^37^, Class II, Class III and Class IV effects^15,23,45^ and acetylcholine-activated potassium current blocking^24^ effects. However, amiodarone has frequent and sometimes serious extracardiac adverse effects^4^ greatly limiting its therapeutic use. This problem, at least partly, can be attributed to its benzofuran structure, since the development of the iodide free amiodarone analogue, dronedarone, has not met the expectations both in respect to efficacy and safety^31^. It is important to note that SZV-2649 like amiodarone, has multiple ion channel blocking properties which could be characterized by a mexiletine-like fast kinetics inhibition of the inward sodium and inward L-type calcium currents. Like amiodarone, SZV-2649 inhibits several types of outward potassium currents including hERG/I_Kr_ and acetylcholine-activated outward potassium currents. It has to be mentioned that SZV-2649 inhibited hERG current more potently than I_Kr_. This can be due to possible species/preparation dependent differences. Also it was previously shown that drug effects in hERG current and in I_Kr_ measurements can substantionally differ^46,47^ since hERG unlike I_Kr_ lacks beta or other accessory ion channel subunits. The observed APD_50_ shortening in Purkinje fibers by SZV-2649 is consistent with its inhibition of I_NaL_, since this current is particularly important in shaping repolarization configuration in Purkinje fibers. The augmented repolarizing prolongation by SZV-2649 in carbachol pretreated atrial muscle argues for a significant inhibition of I_KACh_ although since it was not investigated in this study, its possible M2 receptor blocking effect cannot be ruled out. The multiple ion channel modifying effects were proposed to play an important role in the strong efficacy and reduced proarrhythmic potential of amiodarone compared to other antiarrhythmic drugs that have either selective Class I or Class III antiarrhythmic effects.

In this study, SZV-2649 was found to be effective against in vivo coronary artery occlusion/reperfusion induced ventricular arrhythmias in rats and carbachol and electrical stimulation induced atrial fibrillation in dogs. These effects of SZV-2649 can be explained by its multiple ion channel bloking properties identified in this study.

SZV-2649, similarly to that of amiodarone, has hERG/I_Kr_ and GIRK potassium as well as inward sodium and calcium current inhibitory effects at sub- or low micromolar concentrations and these effects explain its marked Class IB, Class III and weak Class IV antiarrhythmic effects in isolated dog cardiac papillary and atrial muscle preparations., although it does not prolong ventricular repolarization and QT_c_ as much as selective Class III drugs^48,49^. In addition, in dog cardiac Purkinje fibers, SZV-2649 like amiodarone^37,38^ does not change or even shortens APD_50_ and thus cannot be expected to enhance dispersion of repolarization. This is in sharp contrast to the effects of selective Class III drugs like dofetilide, which in addition to prolonging repolarization, also enhance dispersion of repolarization between the ventricle and Purkinje fibers^50^, increasing proarrhythmic risk. This latter effect, like with amiodarone, can be explained by the multiple ion channel modulator property of SZV-2649, i.e. late sodium and L-type calcium channel inhibition by the compound can limit repolarization prolongation caused by potassium channel inhibition.

## Conclusion

Based on the presented experimental data, SZV-2649 posesses antiarrhythmic properties as it was effective against both experimental atrial and ventricular fibrillation. The cellular electrophysiological mechanisms of SZV-2649 are similar to those of amiodarone, i.e. the compound exhibits Class I/B, Class III and Class IV antiarrhythmic effects with strong inhibition of the acethylcholin-activated potassium current. However, in this study, these effects were achieved by application of a compound with an entirely different and novel chemical structure. Therefore, it can be expected that this new compound, SZV-2649, would have similar antiarrhythmic effects as amiodarone but would also be free from the adverse effects seen after chronic amiodarone treatment, at least partly attributed to the benzofuran structure of amiodarone.

## Methods

### Animals

All experiments were carried out in compliance with the *Guide for the Care and Use of Laboratory Animals* (USA NIH publication NO 85 − 23, revised 1996) and conformed to the Directive 2010/63/EU of the European Parliament. The protocols have been approved by the Ethical Committee for the Protection of Animals in Research of the University of Szeged, Szeged, Hungary (approval numbers: I-74-15-2017 and I-74-24-2017) and by the Department of Animal Health and Food Control of the Ministry of Agriculture and Rural Development (authority approval numbers: XIII/3330/2017 and XIII/3331/2017). Beagle dogs were purchased from an experimental animal breeder, Ásotthalom, Hungary (breeder’s authority approval number: XXXV/2018) certified by the Department of Animal Health and Food Control of the Ministry of Agriculture and Rural Development, Hungary. Fifty-five healthy, male Wistar-Hannover rats weighing 250–300 g were purchased from “Toxi-Coop” Toxicological Research Center Zrt. (Budapest, Hungary). All experiments are reported in compliance with the ARRIVE guidelines.

### Synthesis of SZV-2649

**Fig. 9:**
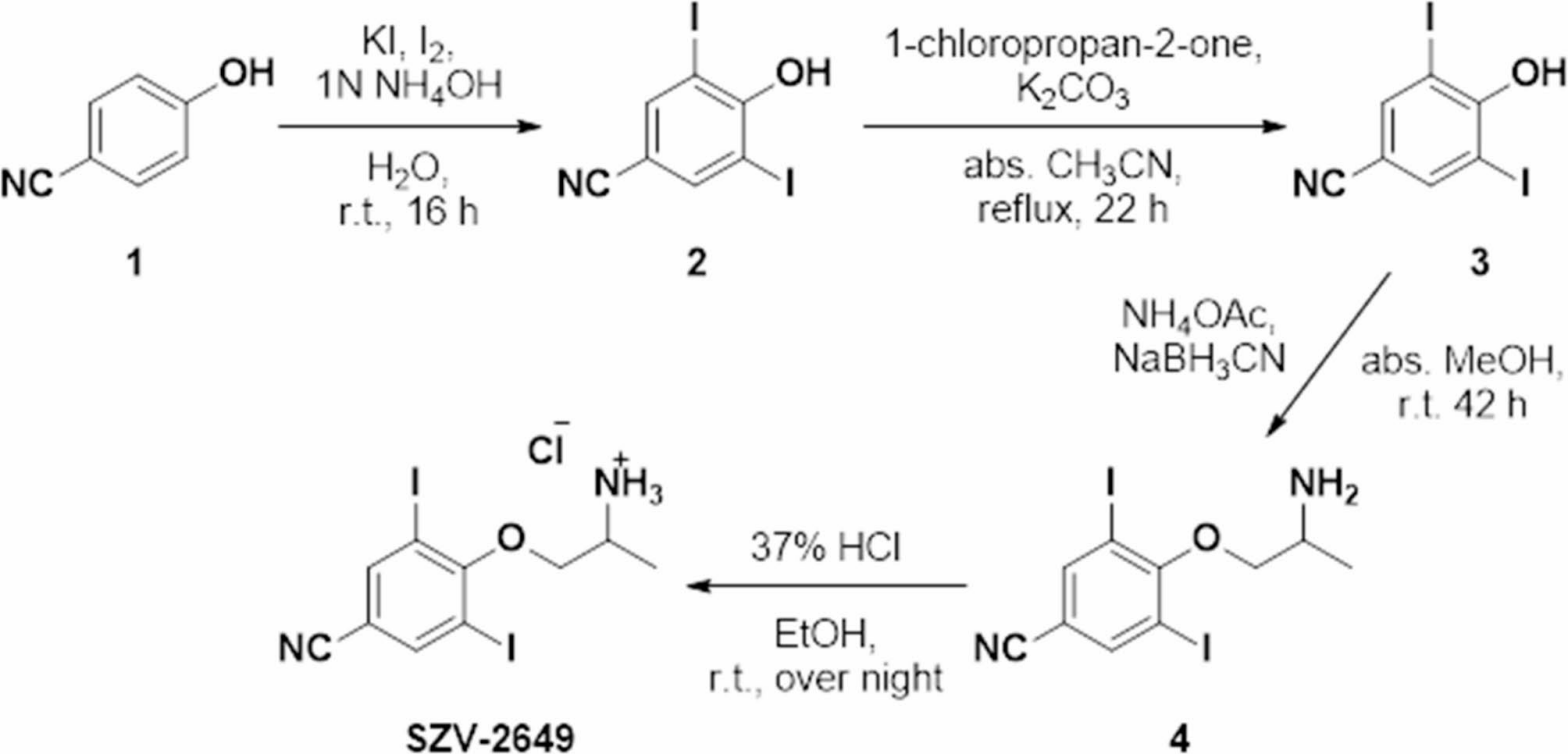
Synthesis of SZV-2649.

For the synthesis of compound **2** recently several methods were published utilizing for example I_2_ in combination with NaClO_3_^51^ or with AcOH and KIO_3_^52^ or with KI and NH_3_^53^ or without the direct use of I_2_ utilizing NaI, H_2_O_2_ and SeCl_4_^54^. In our case method using I_2_, KI and NH_4_OH as NH_3_ source proved to be the best choice as the reaction yielded the product in 92%. As our next step, the oxypropan-2-one moiety could have been formed *via* a Mitsunobu reaction utilizing a phosphine catalyst; however, after several attempts it failed in our case (using Ph_3_P as phosphine derivative and both diisopropyl or diethyl diazodicarboxylate)^55,56^. In the meantime a simple nucleophilic substitution^57–59^ bore success *via* the use of K_2_CO_3_ and chloroacetone in abs. CH_3_CN, under argon atmosphere resulting in compound **3**. The subsequent reductive amination was carried out using NH_4_OAc as amine source and NaBH_3_CN as reducing agent. The reaction was carried out in both EtOH and MeOH showing similar conversions. The final salt formation was carried out using cc. HCl in EtOH yielding SZV-2649 (see Fig. 9).

### Experimental

^1^H and^13^C-NMR spectra were recorded in DMSO-d_6_ and CDCl_3_ solutions in 5 mm tubes at room temperature (r.t.) on a Bruker DRX-500 spectrometer (Bruker Biospin, Karlsruhe, Baden Württemberg, Germany) at 500 (1 H) and 125 (13 C) MHz, with the deuterium signal of the solvent as the lock and TMS as internal standard (1 H, 13 C).

Melting points were determined on a Hinotek X-4 melting point apparatus. Merck Kieselgel 60F_254_ plates were used for TLC.

### 4-Hydroxy-3,5-diiodobenzonitrile (2)

To a solution of 4-hydroxybenzonitrile (2.00 g, 16.79 mmol) in water (400 mL), KI (4.00 g, 24.10 mmol) and I_2_ (8.80 g, 34.40 mmol) were added and the mixture was kept at room temperature. Over a reaction time of 16 h, 1 mL of 1 N NH_4_OH was added dropwise, every half an hour. The reaction was monitored by TLC (*n*-hexane: EtOAc 4:1). After the reaction was complete, Na_2_S_2_O_5_ was added to the system to reduce unreacted I_2_. The aqueous phase was extracted, using DCM (2 × 50 mL). The combined organic phase was dried (Na_2_SO_4_), filtered and concentrated in vacuo. The residue was crystallized using EtOAc (15 mL): 5.70 g white crystal, yield: 92%. The compound has been characterized in various literature, e.g. Patrick et al. (2013)^60^.

### 3,5-diiodo-4-(2-oxopropoxy)benzonitrile (3)

To a solution of 4-hydroxy-3,5-diiodobenzonitrile (5.00 g, 15.00 mmol) in abs. CH_3_CN (50 mL), dry K_2_CO_3_ (10.35 g, 75.00 mmol) and 1-chloropropan-2-one (1.80 mL, d = 1.16 g/mL, 22.60 mmol) were added and the mixture was kept under reflux for 22 h, respectively cooled to room temperature. The inorganic solid was filtered off and washed twice with acetone (2 × 15 mL). After evaporation of the filtrate, the residue was taken up in EtOAc (150 mL) and extracted with 2 N NaOH (4 × 50 mL). The combined organic layer was washed with brine, dried (MgSO_4_), filtered and concentrated in vacuo. The crude product was recrystalyzed from CH_3_CN (50 mL) to give the product: 2.82 g yellow solid, yield: 44%, Mp: 165–167 °C.

HRMS calcd for [M – H^−^] *m*/*z* = 369.8231, found *m*/*z* = 369.8222 (with the decomposition of the 2-oxopropyl- function; Fig. S7, S8); ^1^H (400 MHz, CDCl_3_), δ (ppm): 2.46 (s, 3H), 4.54 (s, 2H), 8.07 (s, 2H), ^13^C (100 MHz, CDCl_3_), δ (ppm): 27.3, 76.1, 90.6, 112.4, 115.0, 143.2, (143.2), 160.6, 203.1. (Figure S1, S2)

### 4-(2-aminopropoxy)-3,5-diiodobenzonitrile (4)

To a cooled solution of 3,5-diiodo-4-(2-oxopropoxy)benzonitrile (2.77 g, 6.50 mmol) in abs. MeOH (30 mL), NH_4_OAc (5.00 g, 65.00 mmol) and NaBH_3_CN (2.05 g, 32.50 mmol) were added and the mixture was stirred 42 h at room temperature. The received suspension was cooled by ice-bath, diluted with H_2_O and the excess of reagent quenched by 37% HCl (pH = 1–2). After adding 4 M NaOH (pH = 12–13), the aqueos phase was extracted with CHCl_3_ (3 × 50 mL). The combined organic layer was dried (MgSO_4_), filtered and concentrated in vacuo to give the product: 2.68 g light yellow solid, yield: 96%, Mp: 110–115 °C.

HRMS calcd for [M + H^+^] *m*/*z* = 428.8955, found *m*/*z* = 428.8947 (Fig. S9, S10);  ^1^H (400 MHz, CDCl_3_), δ (ppm): 1.23 (d, *J* = 6.5 Hz, 3H), 3.49–3.59 (m, 1H), 3.82–3.94 (m, 2 H), 8.06 (s, 2H), ^13^C (100 MHz, CDCl_3_), δ (ppm): 19.7, 47.3, 79.4, 91.0, 111.6, 115.3, 143.2, 195.2. (Figure S3, S4)

### 4-(2-aminopropoxy)-3,5-diiodobenzonitrile hydrochloride (SZV-2649)

The 4-(2-aminopropoxy)-3,5-diiodobenzonitrile (1.25 g, 2.92 mmol) was dissolved in abs. EtOH (20 mL) and cooled by ice-bath. 37% HCl (2.00 mL, d = 1.19 g/mL, 24.00 mmol) was added in small proportions and stirred overnight at room temperature. After concentration in vacuo, the oily residue was triturated with Et_2_O (10 mL), filtered and dried to give the product: 1.13 g white solid, yield: 83%, Mp: 216–219 °C.

HRMS calcd for [M + H^+^] *m*/*z* = 428.8955, found *m*/*z* = 428.8947 (Fig. S11, S12); ^1^H (400 MHz, DMSO), δ (ppm): 1.41 (d, *J* = 6.8 Hz, 3H), 3.64–3.76 (m, 1H), 3.98–4.06 (m, 1H), 4.06–4.13 (m, 1H), 8.24 (brs, 3H), 8.42 (s, 2H), ^13^C (100 MHz, DMSO), δ (ppm): 19.7, 47.3, 79.4, 91.0, 111.6, 115.3, 143.2, 195.2. (Figure S5, S6)

### ECG measurements and the acute atrial fibrillation model in conscious dogs

Beagle dogs of either sex weighing 12–14 kg were used for the experiments. ECG measurements were taken in conscious animals using the standard I-III leads. QT_c_ was calculated using the Van de Water’s formula^61^. SZV-2649 (10 mg/kg) was applied by i.v. or oral administration.

In the atrial fibrillation experiments, at least 5–6 dogs for control and test groups, (respectively) were used. Following 0.5 µg/kg sufentanyl premedication and 150 mg/kg *i.v.* pentobarbital anaesthesia induction, left thoracotomy was performed on all animals. Dogs were endotracheally intubated and mechanically ventilated (UGO Basile respirator; Biological Research Apparatus, Italy) Physiological parameters (non-invasive blood pressure, SpO2, ECG) were continuously monitored during surgery and experiments (InnoCare-VET Patient Care Monitor; Budapest, Hungary). The ECGs were recorded using precordial leads and were digitized and stored on a computer for off-line analysis using National Instruments data acquisition hardware (National Instruments, Austin, Texas, USA) and SPEL Advanced Haemosys software (version 3.2, MDE Heidelberg GmbH, Germany). Under pentobarbital anaesthesia, two pacemaker electrodes (Biotronik Solia S 60; Biotronik Ltd., Hungary) were positioned epicardially into the left atrial appendage and apex of the left ventricle, respectively, and electrodes were connected to pacemakers (Effecta D; Biotronik Hungary Ltd., Hungary). Pacemakers were programmed in VVI mode using Biotronik IC: 4808 A-Renamic programmer to prevent the potentially bradycardic effect of carbachol leading to hemodynamic instability. Atrial and ventricular thresholds were measured before AF induction in all animals. Ventricular and atrial pacing were set to three times the measured threshold. AF inducibility were tested in both groups using a control set (25 times) of 10-second-long rapid atrial bursts (800 beats/min, at threefold threshold). Following 25 atrial burst stimuli, 2 × 2 µg/kg loading dose of intravenous carbachol was administered followed by an 8 µg/kg/h maintenance dose. Atrial fibrillation was induced using 10-second-long atrial burst stimuli in the presence of carbachol in both groups of dogs. In case atrial fibrillation was not converted spontaneously to sinus rhythm, control dogs received 0.5 mg/kg propafenone intravenously in 5 min (as a positive control), while the study group of dogs received the SZV-2649 the 10 mg/kg dose in 5 min. After conversion to sinus rhythm, inducibility of AF was repeatedly tested using 10-second-long atrial burst stimuli in the presence of carbachol and propafenone (control group) and in the presence of carbachol and SZV-2629. In these experiments, we investigated whether AF was converted to SR by the test drug(s). All intravenous infusions were performed using a programmable infusion pump (Terufusion TE-3; Terumo Europe, Leuven, Belgium).

### Coronary artery occlusion/reperfusion-induced ventricular arrhythmias in anesthetized rats

The surgical procedure was based on a modified method described in detail in Gömöri et al. (2020)^62^. Pentobarbital-anesthetized male Wistar-Hannover rats were mechanically ventilated and subjected to thoracic surgery to perform a 6-min coronary artery occlusion and 5-min of reperfusion for the development of ischemia-induced ventricular arrhythmias (see Fig. 6A). The right jugular vein was cannulated for the administration of SZV-2649, vehicle, mexiletine or dofetilide, and the right carotid artery was used to measure blood pressure. The three-lead electrocardiogram and body temperature were continuously registered. Ten to twelve rats per group were administered vehicle (dimethyl sulfoxide, 200 µL/kg) or the studied drugs (see above), 2 min before the onset of ischemia as a slow *i.v.* bolus injection through the right jugular vein. At the end of 5-min reperfusion, hearts were isolated, mounted onto a Langendorff perfusion system, and after re-occluding the coronary artery, were perfused with 5-mL of 70% ethanol via the ascending aorta to delineate the ischemic risk zone. Then the left ventricles were cut along the demarcation lines of denatured area, and the sections were weighed to determine the area of risk to normalize the extent of the ischaemic insult. We evaluated the occurrence of ventricular arrhythmias based on the recommendations of the modified Lambeth Conventions published previously by Curtis et al. (2013)^63^.

### Conventional microelectrode technique

Action potentials were recorded in cardiac ventricular trabeculae and papillary muscle preparations obtained from the right ventricles of Beagle dogs (10–15 kg) of either sex using the conventional microelectrode technique. Dogs were sacrificed by iv. sedation with xylazine (1 mg/kg), followed by i.v. administration of 50 mg/kg pentobarbital. After the cornea reflex disapeared the chest was opened, and the heart was rapidly removed. The heart was immediately rinsed in oxygenated modified Locke’s solution containing (in mM): NaCl 128.3, KCl 4, CaCl_2_ 1.8, MgCl_2_ 0.42, NaHCO_3_ 21.4, and glucose 10. The pH of this solution was set between 7.35 and 7.4 when gassed with the mixture of 95% O_2_ and 5% CO_2_ at 37 °C.

Isolated papillary muscle or atrial trabecula and Purkinje fiber preparations were obtained and prepared from the right ventricle and were individually mounted in a tissue chamber with the volume of 50 ml. Each preparation was initially stimulated through a pair of platinum electrodes in contact with the preparation using rectangular current pulses of 2 ms duration. These stimuli were delivered at a constant cycle length of 1000 ms for at least 60 min allowing the preparation to equilibrate before the measurements were initiated. Transmembrane potentials were recorded using conventional glass microelectrodes, filled with 3 M KCl and having tip resistances of 5–20 MΩ, connected to the input of a high impedance electrometer (Experimetria, type 309, Budapest, Hungary) which was coupled to a dual beam oscilloscope. The resting potential (RP), action potential amplitude (APA), maximum upstroke velocity (*V*_max_), and APD measured at 50% and 90% of repolarization (APD_50_ and APD_90_, respectively) were off-line determined using a software developed in our department (APES) running on a computer equipped with an ADA 3300 analog-to-digital data acquisition board (Real Time Devices, Inc., State College, Pennsylvania) having a maximum sampling frequency of 40 kHz. Stimulation with a constant cycle length of 1000 ms was applied in the course of all experiments. Attempts were made to maintain the same impalement throughout each experiment. In case an impalement became dislodged, adjustment was attempted, and if the action potential characteristics of the re-established impalement deviated by less than 5% from the previous measurement, the experiment continued^64–67^. All measurements were carried out at 37 °C.

### Automated planar patch-clamp measurements

The currents conducted by the hERG and GIRK channels were measured by using planar patch-clamp technology^[,47,68^ in the whole-cell configuration with a four-channel medium-throughput fully automated patch-clamp platform (Patchliner Quattro, Nanion Technologies GmbH, Munich, Germany) with integrated temperature control. Data acquisition and online analysis were performed with an EPC-10 Quadro patch-clamp amplifier (HEKA Elektronik Dr Schulze GmbH, Lambrecht/Pfalz, Germany), using PatchMaster 2.65 software (HEKA Elektronik Dr. Schulze GmbH). The pipetting protocols were controlled by PatchControlHT 1.09.30 software (Nanion Technologies GmbH).

Experiments were carried out at physiological (37 °C) temperature, on HEK293 (human embryonic kidney) cells stably expressing the hERG (Kv11.1) or GIRK1 (Kir3.1/3.4) potassium channels. The cell line originated from Cell Culture Service GmbH (Hamburg, Germany). Cells were cultured at 37 °C, in 5% CO_2_ in IMDM medium (PAA Laboratories GmbH, Pasching, Austria) supplemented with 10% FBS (PAA Laboratories GmbH), 2 mM L-glutamine (Life Technologies Corporation, Carlsbad, California), 1 mM Na-piruvate (PAA Laboratories GmbH), and 500 µg/ml G418 (PAA Laboratories GmbH). Suspension of cells was used for measurements from running cell culture. Cells were washed twice with PBS (Life Technologies Corporation) and then detached with trypsin-EDTA (PAA Laboratories GmbH) for 30–60 s before the measurement. Trypsin was blocked with the serum-containing medium. The cell suspension was next centrifuged (2 min, 100 × g), resuspended in IMDM medium at a final density of 1 × 10^6^ – 5 × 10^6^ cells/ml, and kept in the cell hotel of the Patchliner. Cells were recovered after 15–30 min and remained suitable for automated patch-clamp recordings for up to 4 h.

The following solutions were used during patch-clamp recordings (compositions in mM): internal solution: KCl 50, NaCl 10, KF 60, EGTA 20, HEPES 10, pH 7.2 (KOH); external solution: NaCl 140, KCl 4, glucose-monohydrate 5, MgCl_2_ 1, CaCl_2_ 3, HEPES 10, pH 7.4 (NaOH). All solutions were sterile filtered. Aliquots were stored at − 20 °C and warmed up to room temperature before use. The voltage protocol for hERG ion channel started with a short (100 ms) − 40 mV step to establish the baseline region. A depolarizing step was applied to the test potential of 20 mV for 3 s, and then the cell was repolarized to − 40 mV to evoke outward tail current. Holding potential was − 80 mV. The pulse frequency was 0.1 Hz. Currents were low-pass filtered at 2.9 kHz using the internal Bessel filter of the EPC-10 Quadro patch-clamp amplifier (HEKA Elektronik Dr. Schulze GmbH) and digitized at 10 kHz. The peak tail current was corrected the leak current defined during the first period to − 40 mV. Recording started in external solution. After this control period, 6 increasing concentrations of the test compound were applied, each for approximately 3 min (in case of dofetilide 6 min) to record a complete concentration-response curve. Amitriptyline (10 µM) was applied as a reference inhibitor then a wash-out step terminated the protocol.

For GIRK current measurements (see Fig. 2.), the internal solution was (in mM): K-gluconate 40, NaCl 20, KF 60, EGTA 20 and HEPES 10 (pH 7.2, KOH) supplemented with 0.9 mM GTP before experiments to induce channel activation. External solutions were NaCl 140, KCl 4, glucose monohydrate 5, MgCl 1, CaCl_2_ 3 and HEPES 10, (pH 7.4, NaOH). The high [K^+^] external solution contained NaCl 135, KCl 25, MgCl 1, CaCl_2_ 3 and HEPES 10. The voltage protocol started with depolarizing voltage step to 60 mV for 100 ms before a 0.5 s hyperpolarizing ramp changing to -140 mV. Then the the potential remained at -140 mV for 100 ms before returning to the − 140 mv holding potential. The invard currents were calculated from the − 140 mV segment. The pulse frequency was 0.1 Hz at the beginning of the measurement, the normal 4 mM K^+^ extracellular was changed to high [K^+^] external solution in order to increase the amplitude of the current. After 3 min of control period, the SZV-2649 was added to the cells in increasing concentrations, each for 3 min. Propafenone (1 µM) was used as a reference compound. Finally, K^+^ free external solution was applied to completely inhibit inward K^+^ currents. The measurements were normalized to the recordings taken at K^+^ free external solution serving as baseline.

### Whole cell configuration of the patch-clamp technique

Ventricular myocytes from Beagle dogs were enzymatically dissociated as described in detail previously^49^. A single droplet of cell suspension was placed in a transparent recording chamber mounted on the stage of an inverted microscope (Olympus IX51, Olympus, Tokyo, Japan), and individual myocytes were allowed to settle and adhere to the chamber bottom for at least 5–10 min before superfusion was initiated and maintained by gravity. Only rod-shaped cells with clear striations were used. HEPES-buffered Tyrode’s solution (composition in mM: NaCl 144, NaH_2_PO_4_ 0.4, KCl 4.0, CaCl_2_ 1.8, MgSO_4_ 0.53, glucose 5.5 and HEPES 5.0, at pH of 7.4) served as the normal superfusate.

Micropipettes were fabricated from borosilicate glass capillaries (Science Products GmbH, Hofheim, Germany), using a P-97 Flaming/Brown micropipette puller (Sutter Co, Novato, CA, USA), and had a resistance of 2–3 MOhm when filled with pipette solution. The membrane currents were recorded with Axopatch-200B amplifiers (Molecular Devices, Sunnyvale, CA, USA) by means of the whole-cell configuration of the patch-clamp technique. The membrane currents were digitized with 250 kHz analogue-to-digital converters (Digidata 1440 A, Molecular Devices, Sunnyvale, CA, USA) under software control (pClamp 10, Molecular Devices, Sunnyvale, CA, USA). All patch-clamp experiments were carried out at 37 °C.

#### Measurement of L-type calcium current

The L-type calcium current (I_CaL_) was recorded in HEPES-buffered Tyrode’s solution supplemented with 3 mM 4-aminopyridine. A special solution was used to fill the micropipettes (composition in mM: CsCl 125, TEACl 20, MgATP 5, EGTA 10, HEPES 10, pH was adjusted to 7.2 by CsOH).

#### Measurement of potassium currents

The inward rectifier (I_K1_), the transient outward (I_to_), the rapid (I_Kr_) and the slow (I_Ks_) delayed rectifier potassium currents were recorded in HEPES-buffered Tyrode’s solution. The composition of the pipette solution (mM) was: KOH 110, KCl 40, K_2_ATP 5, MgCl_2_ 5, EGTA 5, HEPES 10 (pH was adjusted to 7.2 by aspartic acid). 1 µM nisoldipine was added to the bath solution to block I_CaL_. When I_Kr_ was recorded, I_Ks_ was inhibited by using the selective I_Ks_ blocker HMR-1556 (0.5 µM). For I_Ks_ measurements, I_Kr_ was blocked by 0.1 µM dofetilide and the bath solution contained 0.1 µM forskolin.

#### Measurement of late sodium current

The late sodium current (I_NaL_) was activated by depolarizing voltage pulses of 2 s at -20 mV from holding potential of -120 mV with pulsing cycle lengths of 5 s. After incubation with the drug for 5 to 7 min, the external solution was replaced with a solution containing 20 µM flecainide, this concentration completely blocked the I_NaL_. The external solution was HEPES-buffered Tyrode’s solution supplemented with 1 µM nisoldipine, 0.5 µM HMR-1556 and 0.1 µM dofetilide in order to block I_CaL_, I_Ks_ and I_Kr_ currents. The composition of the pipette solution (in mM) was: CsCl 125, TEACl 20, MgATP 5, EGTA 10, HEPES 10, pH was adjusted to 7.2 by CsOH).

### Statistics

All data are expressed as means ± SEM. The “*n*” number refers to the number of experiments (i.e. the number of cells in case of patch-clamp and the number of ventricular muscle preparations - papillary or trabecular muscle - in case of action potential measurements). Statistical analysis was performed with paired Student’s *t* test for self-control experiments. The results were considered statistically significant when *P* < 0.05. For the statistical analysis of the incidence of ventricular tachyarrhythmias, the Chi square test with Yates’ corrections was applied, statistical difference was accepted at *P* < 0.05.

## Electronic supplementary material

Below is the link to the electronic supplementary material.

Supplementary Material 1

## Data Availability

The data that support the findings of this study are available from the corresponding author upon reasonable request.
