# Supplementary material for: Effects of SZV-2649, a new multiple ion channel inhibitor mexiletine analogue
Source: Sci Rep. 2024 Oct 5;14:23188. doi: 10.1038/s41598-024-73576-5 (PMC11455950; doi:10.1038/s41598-024-73576-5)
Supplement: Supplementary file 1 — Supplementary Material 1 [file 41598_2024_73576_MOESM1_ESM.doc]

*Supplementary Information*

**Effects of SZV-2649, a new multiple ion channel inhibitor mexiletine analogue**

Aiman Saleh A. Mohammed^1,*^, Muhammad Naveed^1,*^, Tamara Szabados^1^, István Szatmári^2,3^, Bálint Lőrinczi^2^, Péter Mátyus^4,5^, Andrea Czompa^4^, Péter Orvos^1^, Zoltán Husti^1^, Tibor Hornyik^1^, Leila Topal^1^, Szilvia Déri,^1,6^, Norbert Jost^1,6,7^, László Virág^1,7^, Péter Bencsik^1^, István Baczkó^1,7, §,#^, András Varró^1,6,7, §,#^

^1^ Department of Pharmacology and Pharmacotherapy, Albert Szent-Györgyi Medical School, University of Szeged, Szeged, Hungary

^2^ Institute of Pharmaceutical Chemistry, Faculty of Pharmacy, University of Szeged, Szeged, Hungary

^3^ HUN‑REN SZTE Stereochemistry Research Group, Hungarian Research Network, Szeged, Hungary

^4^ Department of Organic Chemistry, Semmelweis University, Budapest, Hungary

^5^ National Laboratory of Infectious Animal Diseases, Antimicrobial Resistance, Veterinary Public Health and Food Chain Safety, University of Veterinary Medicine, Budapest, Hungary

^6^ HUN-REN-SZTE Research Group for Cardiovascular Pharmacology, Hungarian Research Network, Szeged, Hungary

^7^ Interdisciplinary Research and Development and Innovation Centre of Excellence, University of Szeged, Szeged, Hungary

# Corresponding authors:

András Varró, E-mail: [varro.andras@med.u-szeged.hu](../../../../D:%5CPROJECTS%5C_VMA1_2020nov%5CVMA-paper_2022feb%5Cvarro.andras@med.u-szeged.hu).

István Baczkó, E-mail: [baczko.istvan@med.u-szeged.hu](../../../../D:%5CPROJECTS%5C_VMA1_2020nov%5CVMA-paper_2022feb%5Cbaczko.istvan@med.u-szeged.hu)

* Shared first authorship

^§^ Shared senior authorship

**Contents**

1. ^1^H NMR, ^13^C NMR and FTIR spectra of synthesized compounds 2.

2. HRMS spectra of synthesized compounds 5.

3. Representative ECG recordings 8.

3,5-diiodo-4-(2-oxopropoxy)benzonitrile (**3**)


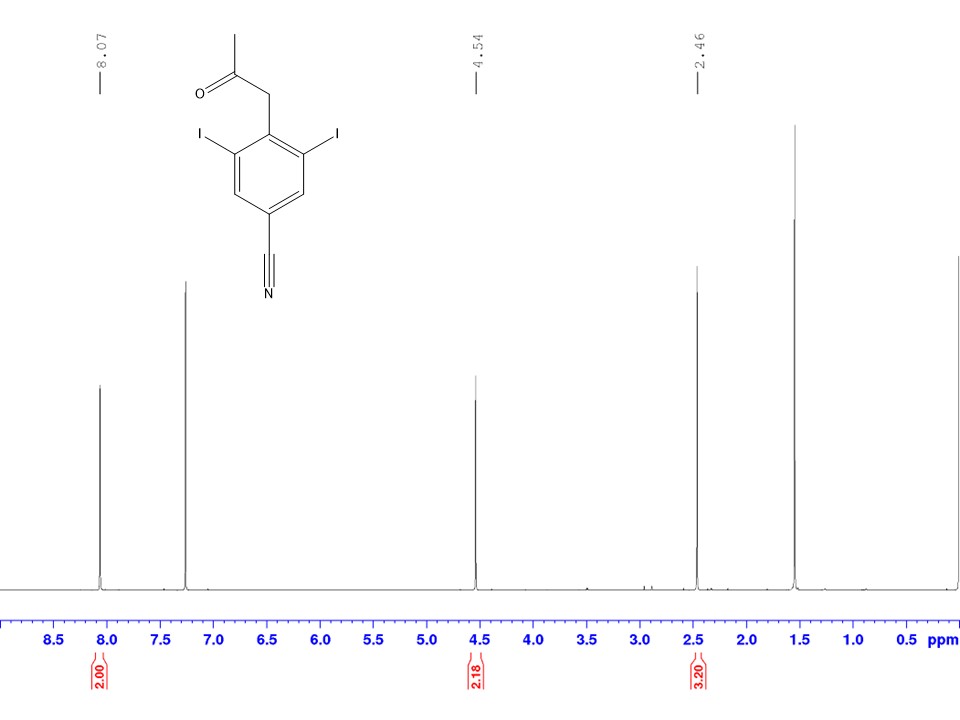


Figure S1. ^1^H-NMR spectrum of **3**


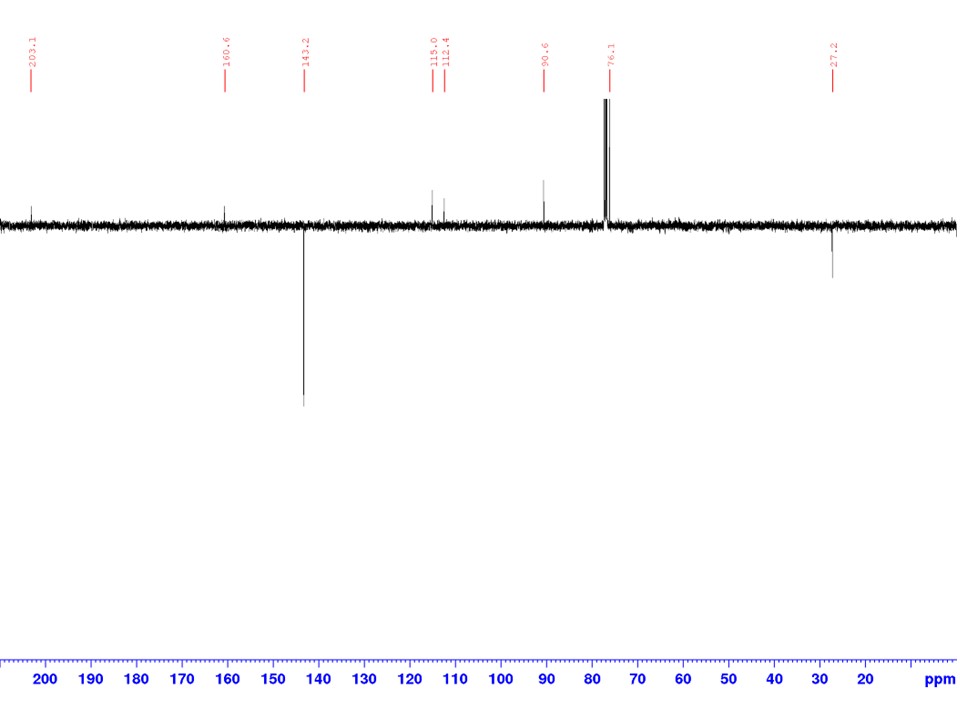


Figure S2. ^13^C-NMR spectrum of **3**

4-(2-aminopropoxy)-3,5-diiodobenzonitrile (**4**)


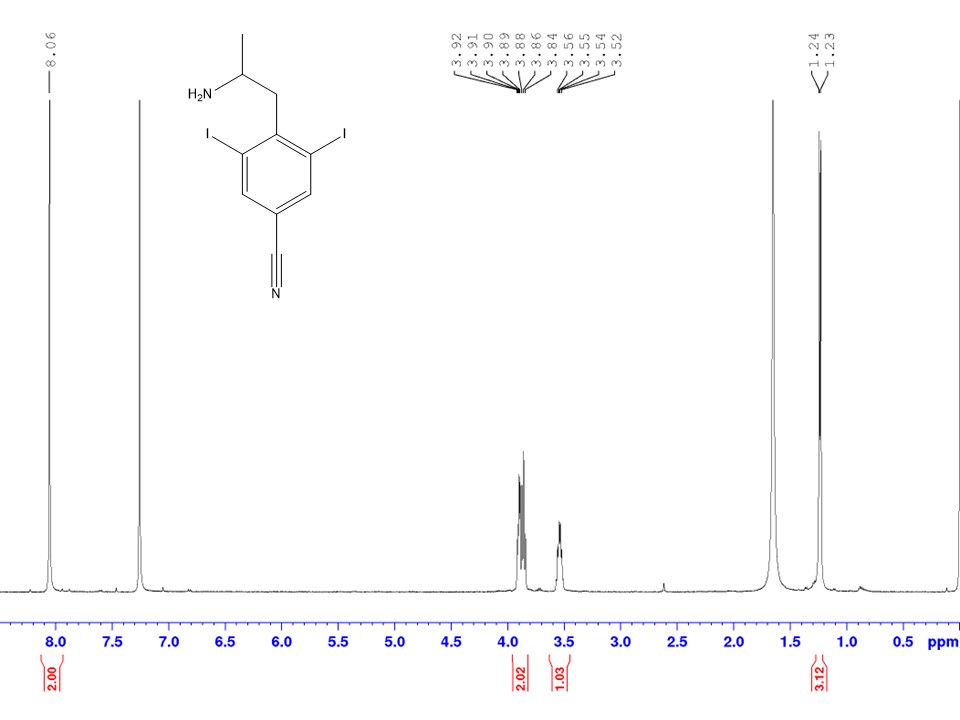


Figure S3. ^1^H-NMR spectrum of **4**


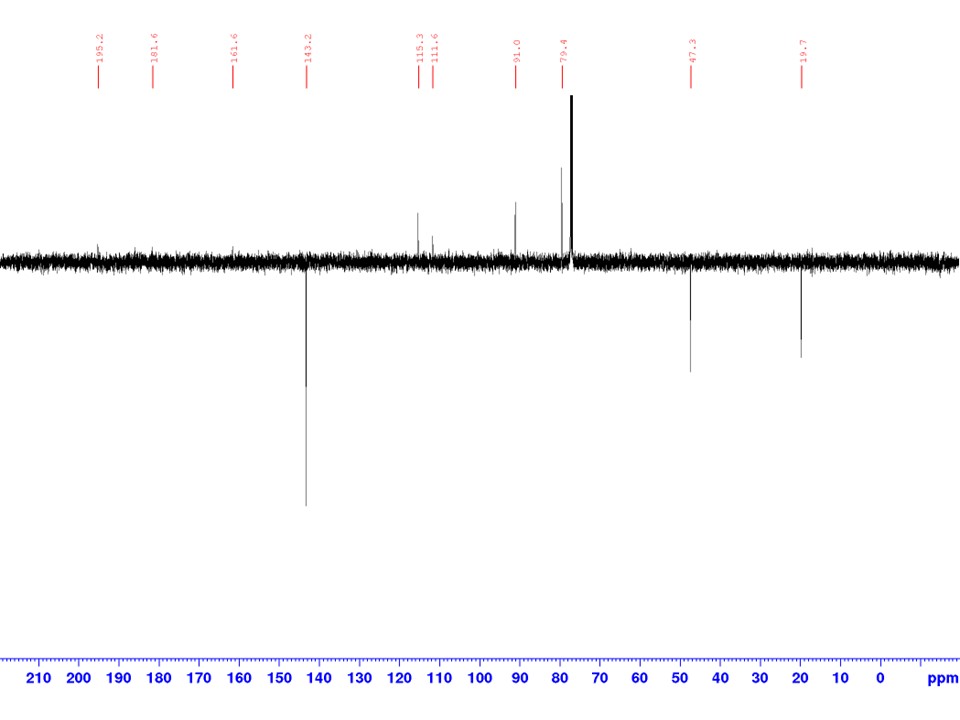


Figure S4. ^13^C-NMR spectrum of **4**

4-(2-aminopropoxy)-3,5-diiodobenzonitrile hydrochloride (**SZV-2649**)


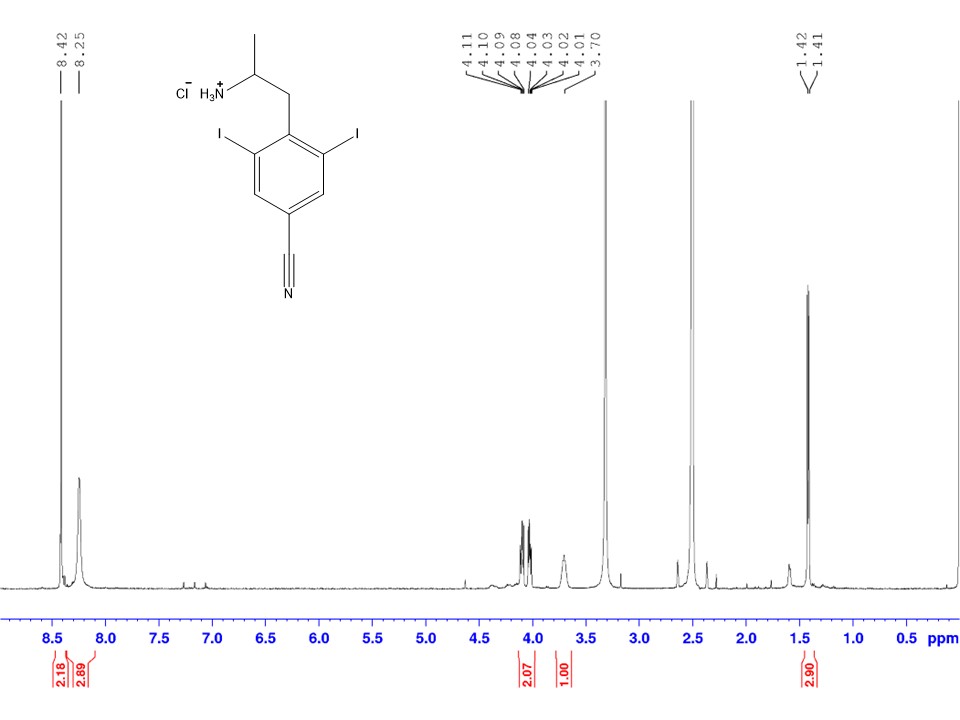


Figure S5. ^1^H-NMR spectrum of **SZV-2649**


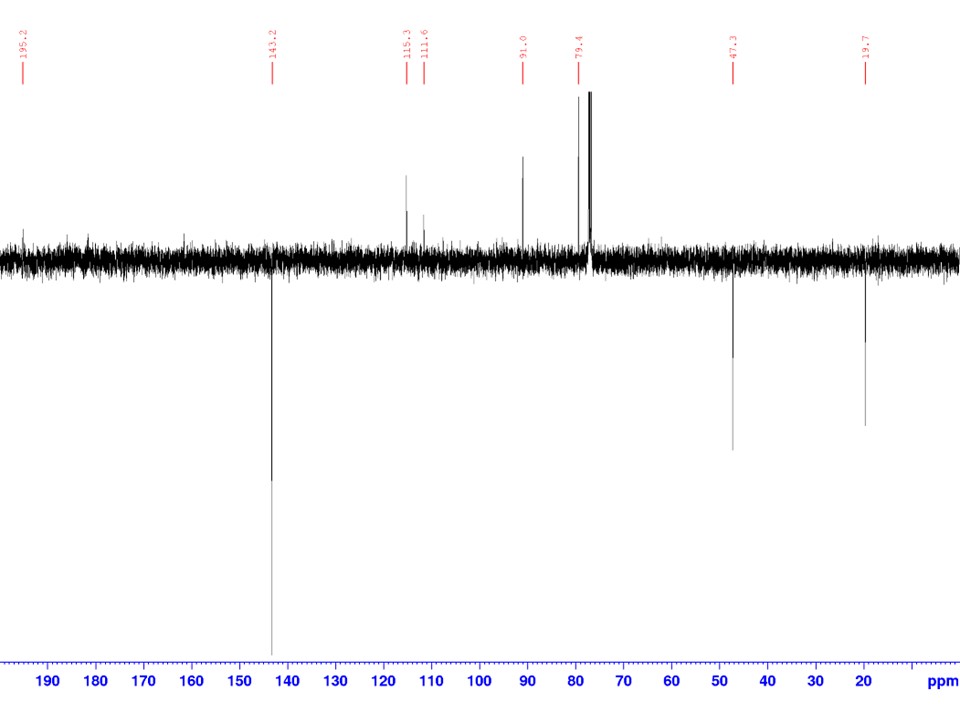


Figure S6. ^13^C-NMR spectrum of **SZV-2649**

3,5-diiodo-4-(2-oxopropoxy)benzonitrile (**3**)


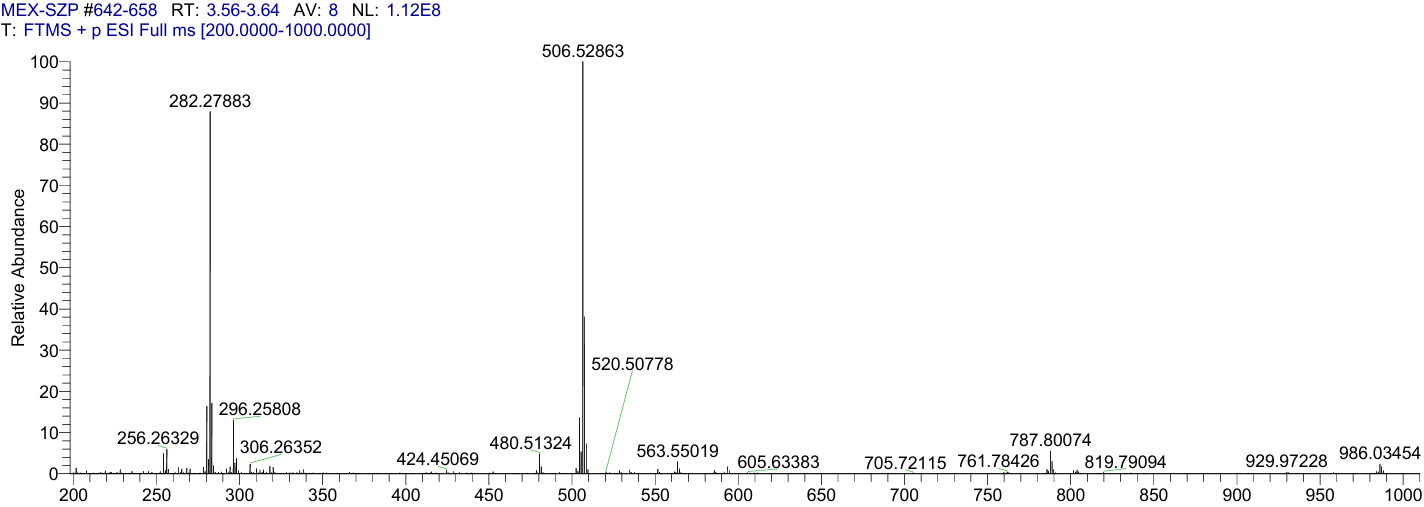


Figure S7. HR-MS (FTMS + p ESI) spectrum of **3**


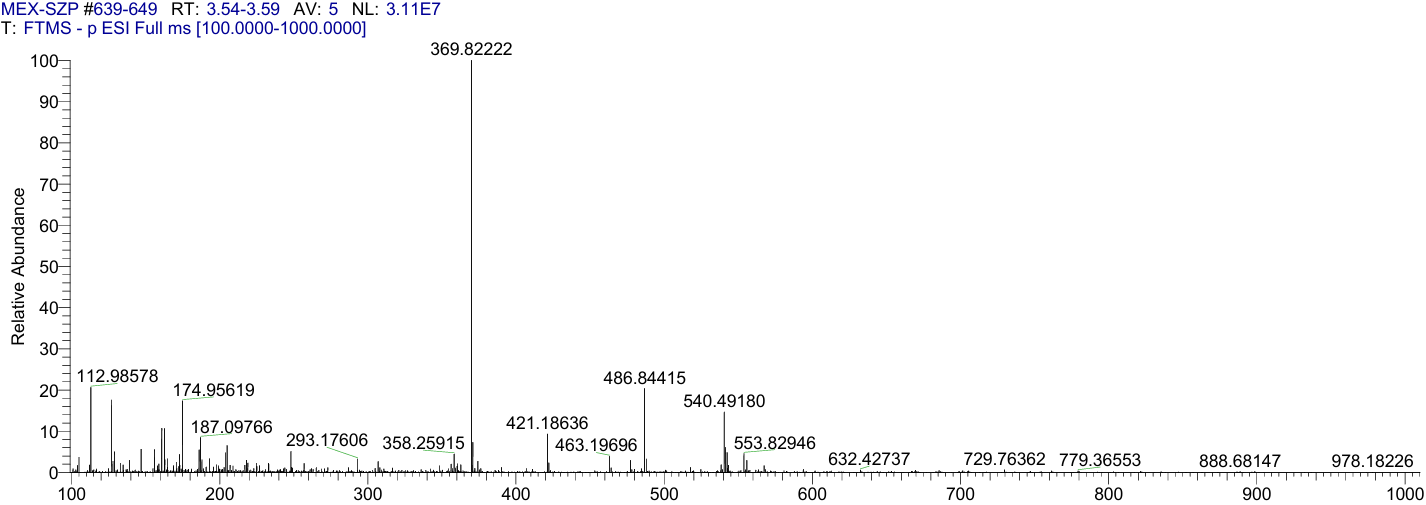


Figure S8. HR-MS (FTMS - p ESI) spectrum of **3**

4-(2-aminopropoxy)-3,5-diiodobenzonitrile (**4**)


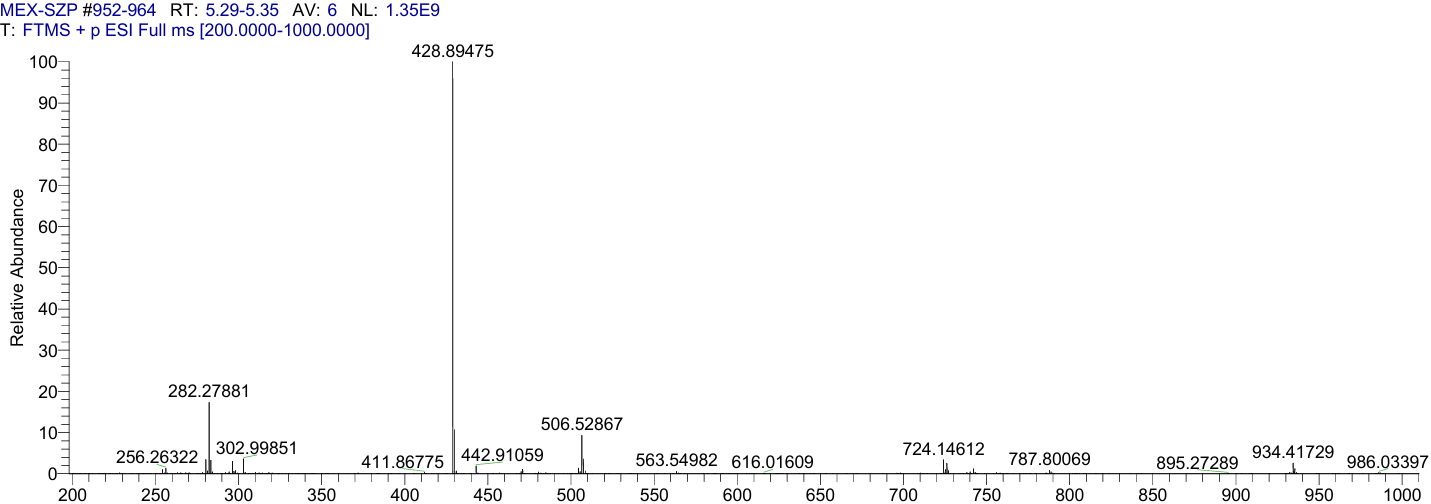


Figure S9. HR-MS (FTMS + p ESI) spectrum of **4**


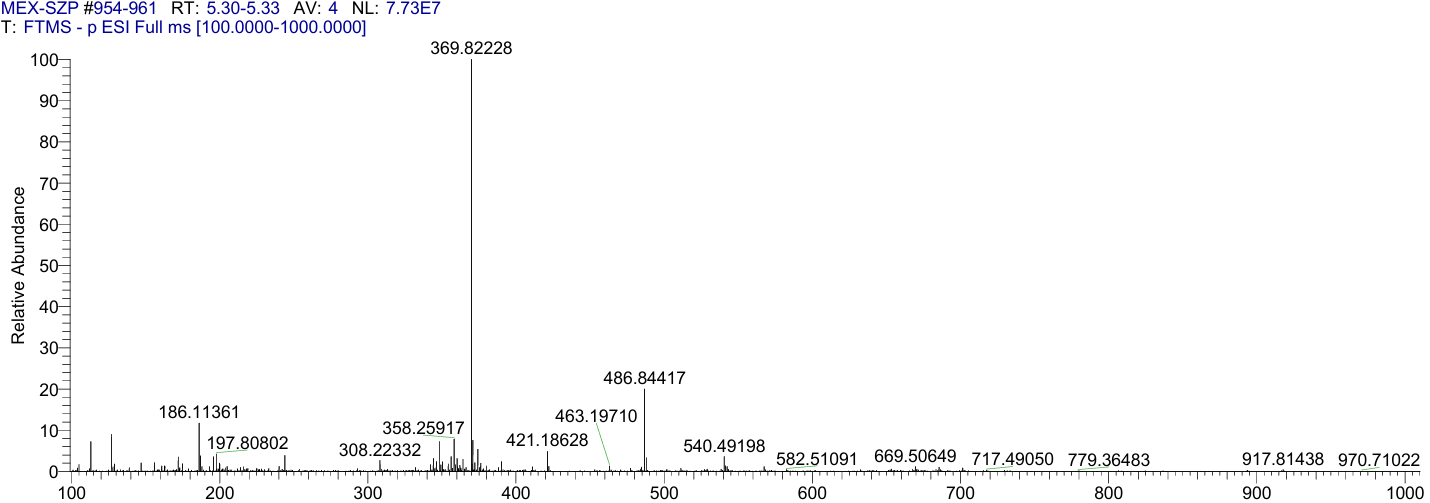


Figure S10. HR-MS (FTMS - p ESI) spectrum of **4**

4-(2-aminopropoxy)-3,5-diiodobenzonitrile hydrochloride (**SZV-2649**)


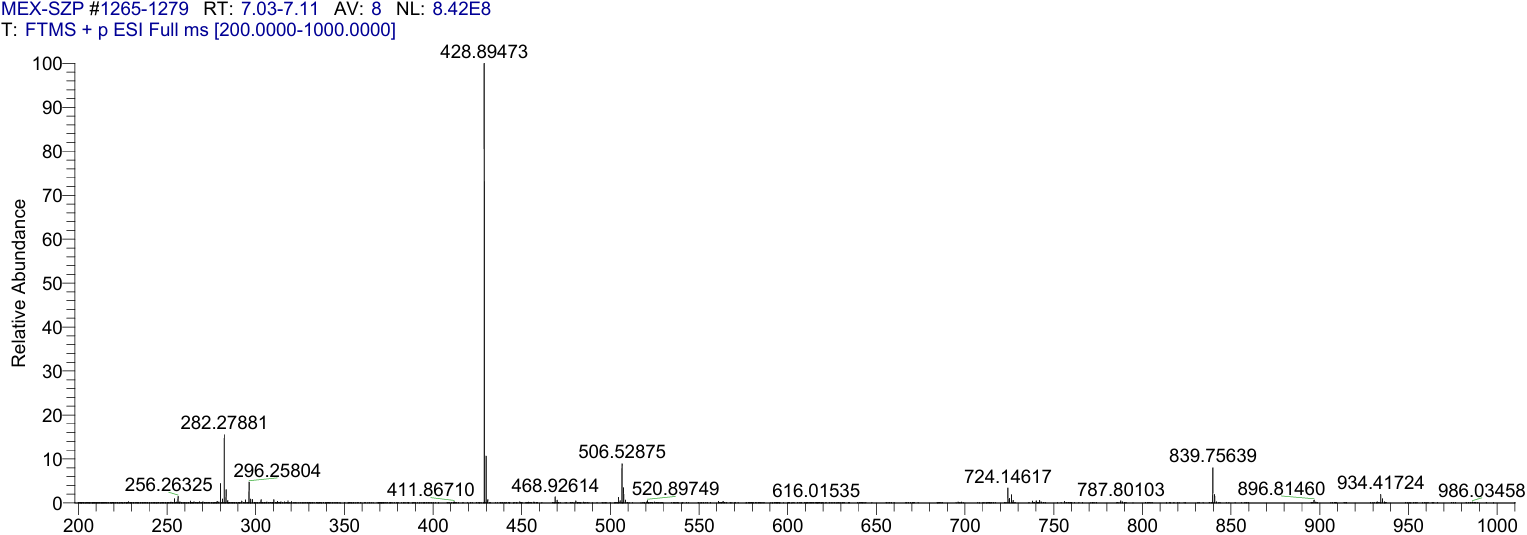


Figure S11. HR-MS (FTMS + p ESI) spectrum of **SZV-2649**


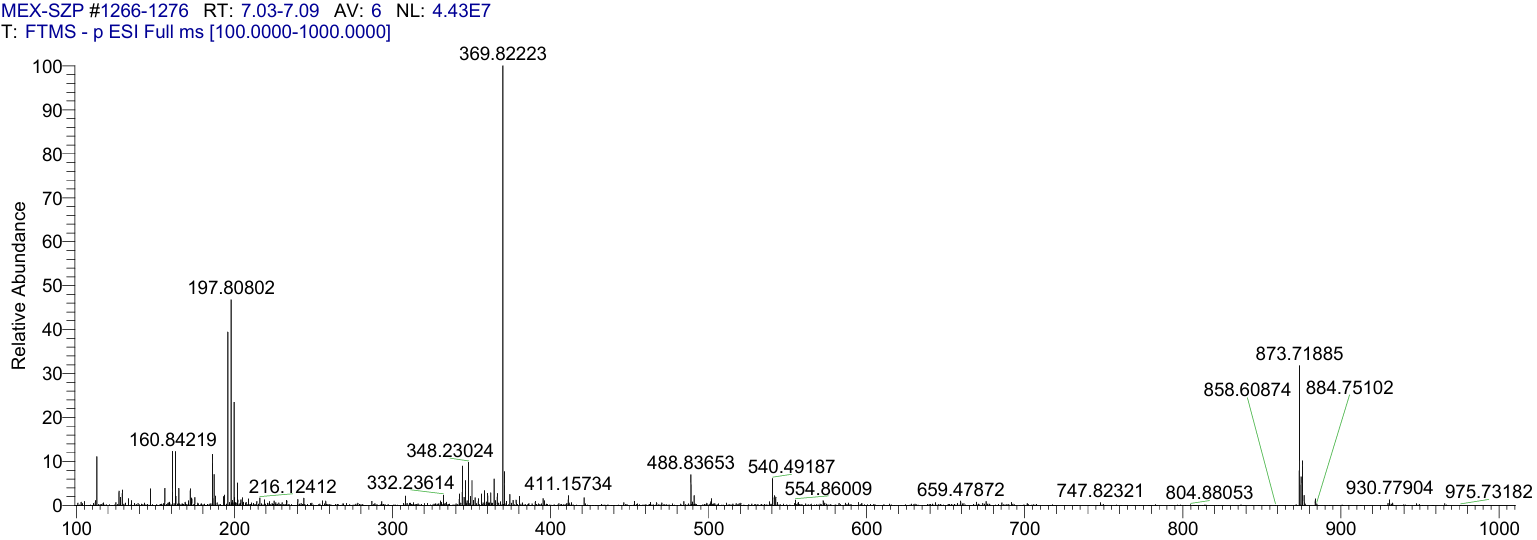


Figure S12. HR-MS (FTMS - p ESI) spectrum of **SZV-2649**


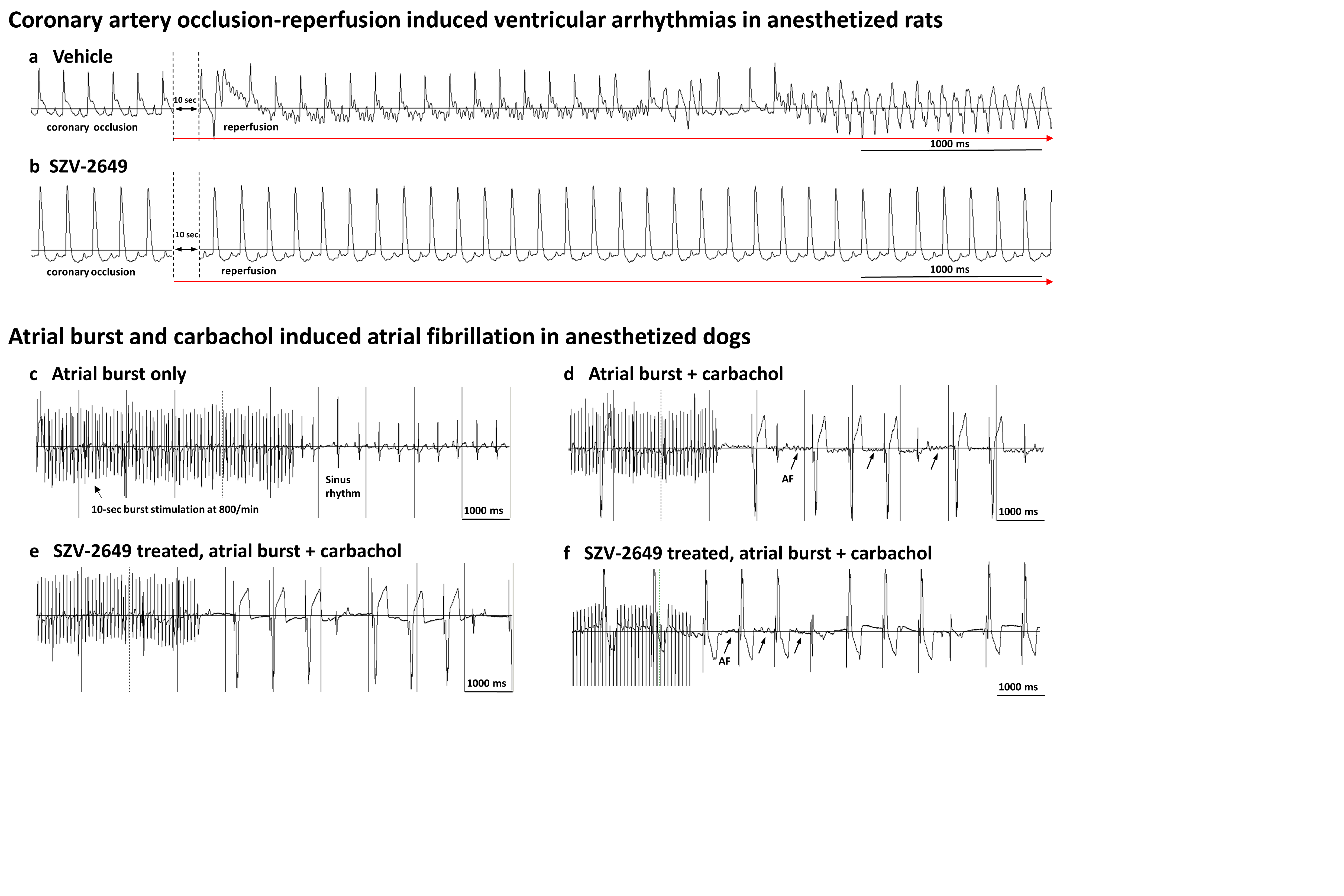


Figure S13. Representative ECG recordings demonstrating the antiarrhythmic effects of SZV-2649. In the coronary artery occlusion-reperfusion induced ventricular arrhythmia anesthetised rat model, the representative vehicle-treated control animal showed early-phase non-sustained ventricular tachycardia shortly after the initiation of reperfusion (**a**), while SZV-2649 administration exhibited protective effect against reperfusion-induced arrhythmia development (**b**). In the atrial burst and carbachol administration induced AF anesthetized canine model, the applied 800/min burst stimulation alone (‚atrial burst only’) could not induce AF (**c**), only when combined with carbachol administration (‚atrial burst + carbachol’) (**d**). SZV-2649 treatment protected against atrial fibrillation as it either completely prevented the burst stimulation+carbachol induced AF development (‚SZV-2649 treated, atrial burst+carbachol) (**e**) or terminated AF within a couple of seconds following the burst stimulus (**f**). Black arrows indicate AF.
